# A Human Mobility-Based Modeling Study of Influenza Vaccination Strategies Across Socioeconomically Disparate Regions in China

**DOI:** 10.3390/vaccines14050425

**Published:** 2026-05-09

**Authors:** Lei Zhou, Yunkang Zhao, Hongjie Wei, Kang Fang, Huimin Qu, Yanshu Ke, Jia Rui, Dayan Wang, Tianmu Chen, Liming Li

**Affiliations:** 1 Department of Epidemiology and Biostatistics, School of Public Health, Peking University, No. 38 Xueyuan Rd, Haidian District, Beijing 100191, China; zhoulei@chinacdc.cn; 2 Chinese Center for Disease Control and Prevention, No. 155 Changbai Road, Changping District, Beijing 102206, China; 3 State Key Laboratory of Vaccines for Infectious Diseases, Xiang An Biomedicine Laboratory, State Key Laboratory of Molecular Vaccinology and Molecular Diagnostics, National Innovation Platform for Industry-Education Integration in Vaccine Research, School of Public Health, Xiamen University, No. 4221-117 South Xiang’an Road, Xiang’an District, Xiamen 361102, China; 32620250156571@stu.xmu.edu.cn (Y.Z.); weihj1998@stu.xmu.edu.cn (H.W.); kfang01@stu.xmu.edu.cn (K.F.); 15223858620@163.com (H.Q.); keyanshu@stu.xmu.edu.cn (Y.K.); ruijia5345@163.com (J.R.); 4 National Institute for Viral Disease Control and Prevention, Chinese Center for Disease Control and Prevention, No. 155 Changbai Road, Changping District, Beijing 102206, China; wangdayan@ivdc.chinacdc.cn; 5 Peking University Center for Public Health and Epidemic Preparedness and Response, No. 38 Xueyuan Rd, Haidian District, Beijing 100191, China; 6 Key Laboratory of Epidemiology of Major Diseases (Peking University), Ministry of Education, No. 38 Xueyuan Rd, Haidian District, Beijing 100191, China

**Keywords:** transmission dynamics model, influenza disease burden, vaccination strategy, differentiated intervention

## Abstract

**Background:** China‘s influenza vaccination coverage remains at a low rate, with significant regional socioeconomic disparities, lacking targeted distribution strategies and achievable coverage targets. This study aims to provide scientific evidence for formulating differentiated and feasible vaccination strategies across Chinese provinces based on regional economic gradients. **Methods:** We employed the Susceptible-Vaccinated-Exposed-Asymptomatic-Infectious-Critical-Fatal-Recovered/Removed (SVEAICFR) model to simulate various vaccination strategies, analyzing the reduction in disease burden and vaccine dose requirements across underdeveloped, developing, and developed regions. The optimal strategy and achievable coverage targets were subsequently determined. **Results:** The 31 provinces were clustered into three categories based on economic levels, showing significant spatiotemporal differences in epidemics (Kruskal–Wallis test, all *p* < 0.001). Developed regions showed the earliest onset and highest peaks (influenza-like illness positive (ILI+) index ≈ 12–13, Baidu Influenza Search Index (BISI) ≈ 310,000). Developing regions exhibited moderate lagging by 1–2 weeks, while underdeveloped regions had the lowest peaks (ILI+ 3–4) and longer epidemic cycles. During the 2023–2024 influenza season, the national predicted vaccination rate was only 2.89% with marked regional disparities. Baseline incidence, severity, and mortality rates were 13,374.93, 49.52, and 8.37 cases per 100,000 population, respectively. Modeling indicates that increasing influenza vaccination coverage rates for populations aged <18 and ≥65 to a theoretical threshold (39.73% of the total population) before the season could reduce incidence, severity, and mortality rate by 99.26%,99.42%, and 99.46%, respectively. **Conclusions:** Influenza prevalence in China exhibits significant regional heterogeneity, necessitating differentiated measures based on regional economic gradients. Regional support mechanisms should be implemented to promote equitable vaccine distribution. Priority vaccination for high-risk populations (aged <18 and ≥65), to reach a 40% theoretical national coverage target, is recommended via realistic implementation pathways to minimize the disease burden of influenza.

## 1. Introduction

Influenza is a pervasive acute respiratory infectious disease with a significant global impact [1]. Annually, seasonal influenza results in approximately one billion cases worldwide, including 3 to 5 million severe infections that cause between 290,000 and 650,000 deaths [2]. In China, which has a population exceeding 1.4 billion and features a rapidly aging demographic and high mobility, the burden of influenza is substantial, especially among older adults [3]. On average, influenza-like illnesses account for roughly 3 million emergency department visits and 2.34 million hospitalizations for severe acute respiratory infections each year in the country [4]. From 2011 to 2019, influenza caused annual economic losses in China estimated between 33 and 106 billion Chinese yuan (CNY) [4]. Although stringent public health and social measures (PHSMs)—including universal masking, physical distancing, school closures, and international travel restrictions—temporarily suppressed influenza transmission by minimizing population contact and respiratory droplet exposure [5,6], a notable resurgence in incidence was observed following the relaxation of these measures, particularly from February to April 2023 [7]. Since November 2023, a surge in cases has been reported in several provinces, including Beijing and Liaoning in northern China, with a pronounced increase among children, underscoring the urgent need for enhanced preventive and control strategies [8].

Vaccination represents one of the most effective and cost-efficient strategies for preventing influenza and mitigating its associated disease burden [9,10]. Vaccine effectiveness (VE) can attain 60% when the vaccine antigen closely matches circulating viral strains, although the average VE is approximately 38% [11]. Influenza vaccination significantly reduces disease severity, complications, and mortality across various age groups [12]. It demonstrates particularly high effectiveness in children aged 6 months to 7 years. Among individuals aged over 65 years, the vaccine is effective in preventing severe complications, contributing to a 4.6% (95% CI: 0.7–8.3%) reduction in all-cause mortality and an 8.5% (95% CI: 3.3–13.5%) decrease in pneumonia- and influenza-related hospitalization rates [13,14]. These benefits underscore the critical importance of well-planned vaccination strategies for effective influenza control.

Despite its proven benefits, influenza vaccination coverage in China has remained persistently low. The overall vaccination rate was 9% in 2011 [15], declining to merely 2.47% for the general population in 2022 [16]. This is largely attributed to the self-paid vaccination system in most provinces. However, coverage among older adults is notably higher, ranging from 30% to 45%, primarily because a handful of relatively wealthy cities provide free influenza vaccination for older adults funded by local governments [17]. Despite this regional policy support, it remains substantially below the World Health Organization (WHO) targets [18]. This low uptake fosters suboptimal herd immunity, increasing population vulnerability to outbreaks and complicating public health control efforts. A fundamental barrier is the exclusion of the influenza vaccine from China’s National Immunization Program. Although free vaccination initiatives have been introduced in 256 districts and counties, eligibility remains restricted, covering only a small fraction of the population [16,19]. The annual growth in vaccination coverage has consistently been below 3%, exacerbating the public health burden [19]. A stark disparity exists between free and self-paid vaccination groups; during the 2020–2021 period, coverage was approximately 38.32% in free vaccination groups compared to just 1.81% in self-paid groups [16].

Socioeconomic factors, divergent government priorities, and varying capacities of vaccination services have collectively created a highly heterogeneous landscape of vaccination coverage across different provinces. By 2018, 11 economically prosperous cities had incrementally implemented government-subsidized vaccination programs targeting high-risk groups, such as older adults and students, achieving an average coverage of 35.40% within these groups [20]. This stands in stark contrast to the national average of 2.47% recorded in 2022 [16]. A national study on influenza vaccination coverage from 2014 to 2021 further highlighted significant regional variation, with rates ranging from 0.77% to 6.69% [21]. The highest coverage was reported in Xinjiang, Tianjin, and Shanghai (6.69%, 6.17%, and 5.56%, respectively), whereas the lowest rates were observed in Xizang, Jilin, and Hainan (0.77%, 1.03%, and 1.04%, respectively) [21]. These disparities are closely linked to regional economic development, fiscal capacity for public health subsidies, and accessibility of healthcare services. They underscore how targeted, well-resourced vaccination strategies can significantly enhance coverage and alleviate disease burden. Integrating influenza vaccines into the national immunization schedule and expanding eligibility for free vaccination could substantially improve influenza control in China.

Conducting real-world trials to evaluate vaccination strategies is challenging, especially in a vast and heterogeneous country like China. Consequently, numerous studies have employed mathematical modeling to simulate the impact of various vaccination strategies on influenza burden [22,23,24,25], demonstrating their utility for theoretical assessment of strategy efficacy [26]. Our study divides China’s provinces into different clusters based on their socioeconomic development levels and analyzes the epidemiological distribution patterns of time, space, and population within each cluster. The reliability of the analytical framework is ensured through double validation using the influenza-like illness positive (ILI+) index and Baidu Influenza Search Index (BISI). On this basis, we simulate various vaccination schemes and systematically evaluate their effectiveness, aiming to identify optimal vaccination strategies across three key dimensions: timing, geographic regions, and target populations. The aim is to quantitatively evaluate the differential impacts of various vaccination strategies on disease burden across socioeconomic clusters, thereby generating evidence-based information that can support the future formulation of national vaccination targets and the optimization of vaccination strategies.

## 2. Materials and Methods

### 2.1. Study Design

This study takes the 2023–2024 influenza season in China as a case study, during which A(H3N2) and B/Victoria line variants exhibited alternating epidemic trends [2]. Our objective is to evaluate and optimize influenza vaccination strategies tailored to provinces with varying levels of economic development, along with their implementation pathways. In this context, “optimization” involves constructing a mathematical model based on the natural history of influenza, building upon the actual epidemic status (double-validated by ILI+ and BISI) and current vaccination coverage rates [15,17]. The study simulates diverse scenarios across temporal, spatial, and population dimensions to propose regionalized intervention mechanisms to bridge the gap between current vaccination levels and identified targets.

The study design is divided into several key phases. Initially, relevant data, including influenza-like illness (ILI) surveillance data (2014–2023) from the Chinese Center for Disease Control and Prevention (China CDC, Beijing, China), and demographic and provincial-level socioeconomic indicators—specifically per capita Gross Domestic Product (GDP)—were obtained from the National Bureau of Statistics of China (Beijing, China), and the population mobility data and search index were collected from Baidu, Inc. (Beijing, China). The Moving Epidemic Method (MEM) is then applied via Python 3.9 (Python Software Foundation, Beaverton, OR, USA) to ILI surveillance data to accurately determine the start and end of the influenza season for each province, thereby defining the precise simulation period for subsequent modeling analyses [27]. The MEM establishes a baseline and epidemic threshold value utilizing historical pre-epidemic and post-epidemic ILI data, we calculated the epidemic threshold for each region. Consistent with established protocols, the upper limit of the 95% Confidence Interval (95% CI) of the historical baseline was defined as the epidemic onset threshold. The temporal simulation window was rigidly bound by the earliest onset and the latest termination date across all provincial configurations. Recognizing the significant economic disparities across China, the 31 provinces are categorized into distinct clusters based on their per capita GDP data. An initial epidemiological description was conducted to analyze spatiotemporal variations in demographics, influenza prevalence, and vaccination coverage across the clusters. Following the precise definition of the epidemic period and spatial clusters, an extended, compartmental mathematical model—Susceptible-Vaccinated-Exposed-Asymptomatic-Infectious-Critical-Fatal-Recovered (SVEAICFR)—was developed to reconstruct the underlying transmission dynamics. Grounded in the natural history of influenza, the model fundamentally incorporates distinct age-specific contact matrices (<18, 18–64, and ≥65 years old), multi-level population mobility structures, and dynamic heterogeneous vaccination profiles. The core mechanistic parameters considered within the SVEAICFR framework include transmission rate (*β*), the proportions of asymptomatic cases, as well as severe and fatality outcomes, the incubation (latent) period and recovery period of the cases (for detailed information see Section 2.3). This model was validated using two key metrics and calibration methods. Following successful validation, the model was employed to simulate a range of vaccination scenarios. These simulations aimed to evaluate the effectiveness of each strategy in reducing the disease burden. By comparing the outcomes of these simulations, we identified the optimal vaccination strategy (for detailed information see Section 2.4).

### 2.2. Data Collection

In this study, a range of data sources is used to capture demographic, economic, epidemiological, and behavioral indicators. Annual demographic data and per capita GDP figures for each province were extracted from the China Statistical Yearbook 2024, published by the National Bureau of Statistics ((https://www.stats.gov.cn/sj/ndsj/) (accessed on 5 February 2025). Provincial influenza surveillance data, including weekly influenza positivity rates and the percentage of influenza-like illness (ILI%) consultations, were obtained from the Chinese Center for Disease Control and Prevention (China CDC) for the period from January 2023 to April 2024. Historical annual influenza vaccination coverage rates from 2014 to 2022 for each province were obtained from the China CDC reports and relevant published studies [16,21]. To assess public awareness and behavior related to influenza, the daily Baidu Influenza Search Index (BISI) for each province between 2023 and 2024 is collected using the Baidu Index platform (http://index.baidu.com) (accessed on 3 January 2025). The BISI is constructed using search queries for the keywords: ‘flu’, influenza’, ‘Influenza A’, and ‘Influenza B’. Additionally, to quantify population mobility, the daily China Baidu Migration Index (CBMI) for each province during the same period is collected from the Baidu Map Migration Big Data platform (https://qianxi.baidu.com/#/) (accessed on 1 January 2025).

### 2.3. Model Construction and Parameter Estimation

To capture the complex transmission dynamics of the 2023–2024 influenza season—specifically the alternating prevalence of A(H3N2) and B/Victoria lineages—we extended the classical Susceptible-Exposed-Asymptomatic-Infectious-Recovered/Removed (SEIAR) compartmental framework. Incorporating vaccination status, clinical severity, and spatial metapopulation dynamics, we developed a deterministic Susceptible-Vaccinated-Exposed-Asymptomatic-Infectious-Critical-Fatal-Recovered/Removed (SVEAICFR) model [28].

**Figure 1:**
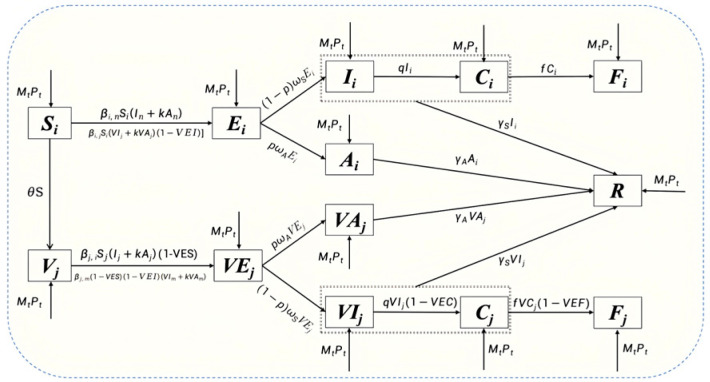
Flowchart of the SVEAICFR mathematical model.

In this model, the population is primarily stratified by vaccination status (unvaccinated vs. vaccinated) and age. Specifically, let *i*, *j* denote the vaccination status corresponding to unvaccinated individuals and vaccinated individuals. For each vaccination status, the population is partitioned into three distinct age groups: under 18 years, 18–64 years, and ≥65 years, respectively (where under 18 years and ≥65 years represent high-risk demographics). Within each stratum, the population is partitioned into distinct epidemiological compartments: susceptible (*S*), vaccinated (*V*), exposed (*E*, *VE*), symptomatic infected (*I*, *VI*), critically infected (*C*, *VC*), asymptomatic infected (*A*, *VA*), fatalities (*F*), and recovered individuals (*R*). The model structure is illustrated in Figure 1.

The mathematical model relies on the following core assumptions:

- Homogeneous Mixing: We assume a well-mixed population within each geographic unit, characterized by random interactions proportional to the contact matrix across age strata.
- Spatial Metapopulation Dynamics: To incorporate spatial heterogeneity, the model accounts for the daily influx and outflux of individuals ($M_{t}$) across provinces. We introduce metapopulation transfer rates to denote the adjusted proportion of the population occupying each specific epidemiological compartment post-migration ($P_{t}$).
- Force of Infection: Transmission is driven by contact between susceptible individuals and infectious individuals (both symptomatic and asymptomatic). The baseline transmission rate is denoted by β. To account for differential infectivity, the infectiousness of asymptomatic cases is scaled by a relative transmissibility coefficient transmissibility rate of asymptomatic to symptomatic individuals, k (yielding an effective rate of kβ).
- Disease Progression: Upon infection, individuals enter an incubation or latent period. A proportion p∈(0,1) of these exposures progress to asymptomatic infection, while 1−p progress to symptomatic disease. The relative transition rates from the incubation (latent) phase to the infectious phase are inversely proportional to their respective incubation (latent) periods ($1/\omega_{s}$ for symptomatic infections and $1/\omega_{A}$ for asymptomatic infections).
- Recovery and Clinical Outcomes: The recovery rates are given by $\gamma_{s}$ and $\gamma_{A}$ for symptomatic and asymptomatic cases, respectively. We assume that only symptomatic infections have the potential to escalate to severe clinical outcomes. The probability of progressing to critical illness is given by q, and the condition-specific case fatality ratio among critically ill patients is denoted by f.
- Vaccine Effectiveness (VE): Using an influenza vaccination coverage parameter n, vaccination operates as a multifaceted intervention. The vaccine induces leaky immunity, reducing susceptibility by a factor of 1−VES, infectiousness by 1−VEI, severe disease risk by 1−VEC, and mortality by 1−VEF.
- Temporal Dynamics: In sequential vaccination scenarios, individuals transition continuously from the susceptible compartment (S) to the vaccinated compartment (V) at a vaccination rate θ. Given the short temporal horizon of a single influenza season, vital dynamics (natural births and baseline mortality) are assumed to be negligible and are excluded from the system.

The transmission dynamics are governed by the following system of ordinary differential equations (ODEs):

(1) $$\frac{{dS}_{i}}{dt}=-\beta_{i}S_{i}(I_{i}+kA_{i})-\beta_{ij}S_{i}({VI}_{j}+k{VA}_{j})(1-V{EI}_{j})-\theta S_{i}+M_{t}P_{t}$$

(2) $$\frac{dE_{i}}{dt}=\beta_{i}S_{i}(I_{i}+kA_{i})+\beta_{ij}S_{i}({VI}_{j}+k{VA}_{j})(1-V{EI}_{j})-{p\omega }_{A}E_{i}-{\left(1-p\right)\omega }_{S}E_{i}+M_{t}P_{t}$$

(3) $$\frac{dI_{i}}{dt}={\left(1-p\right)\omega }_{S}E_{i}-\gamma_{s}I_{i}-qI_{i}+M_{t}P_{t}$$

(4) $$\frac{dA_{i}}{dt}={p\omega }_{A}E_{i}-\gamma_{A}I_{i}+M_{t}P_{t}$$

(5) $$\frac{dC_{i}}{dt}=qI_{i}-\gamma_{s}C_{i}-fC_{i}$$

(6) $$\frac{{dF}_{i}}{dt}=fC_{i}$$

(7) $$\frac{{dR}_{i}}{dt}={\gamma_{s}I_{i}+\gamma }_{A}A_{i}+M_{t}P_{t}$$

(8) $$\frac{{dV}_{j}}{dt}=-\beta_{ji}S_{j}(I_{i}+kA_{i})(1-VES_{j})-\beta_{j}S_{j}(1-V{ES}_{j})(1-V{EI}_{j})({VI}_{j}+k{VA}_{j})+\theta S_{i}+M_{t}P_{t}$$

(9) $$\frac{d{VE}_{j}}{dt}=\beta_{ji}S_{j}\left(I_{i}+kA_{i}\right)\left(1-VES_{j}\right)+\beta_{j}S_{j}\left(1-V{ES}_{j}\right)\left(1-V{EI}_{j}\right)\left({VI}_{j}+k{VA}_{j}\right)-{p\omega }_{A}VE_{j}-\;{(1-p)\omega }_{S}VE_{j}+M_{t}P_{t}$$

(10) $$\frac{d{VI}_{j}}{dt}={(1-p)\omega }_{S}{VE}_{j}-\gamma_{s}{VI}_{j}+M_{t}P_{t}$$

(11) $$\frac{d{VA}_{j}}{dt}={p\omega }_{A}{VE}_{j}-\gamma_{A}VI_{j}+M_{t}P_{t}$$

(12) $$\frac{dVC_{j}}{dt}=q{VI}_{j}(1-V{EC}_{j})-f{VC}_{j}(1-V{EF}_{j})$$

(13) $$\frac{d{VF}_{j}}{dt}=fVC_{j}(1-V{EF}_{j})$$

(14) $$\frac{{dVR}_{j}}{dt}={\gamma_{s}VI_{j}+\gamma }_{A}{VA}_{j}+M_{t}P_{t}$$

(15) $$N=S_{i}+E_{i}+I_{i}+A_{i}+C_{i}+F_{i}+R_{i}+V_{j}+{VE}_{j}+{VI}_{j}+{VA}_{j}+VC_{j}+VF_{j}+{VR}_{j}$$

The explicit definitions, estimated values, and bounds of the model parameters are detailed in Table 1.

### 2.4. Simulation Method and Statistical Analysis

The initial number of cases incorporated into the model is estimated based on our prior research [28], which utilized BISI, population counts, and previously established influenza attack rates for both Northern and Southern China [58]. The specific estimation formula is as follows:

(16) $$I_{i}=\frac{Ypi*coef}{\sum Y_{BISI}}*{BISI}_{i}$$

where $I_{i}$ represents the estimated daily number of influenza cases in the province, Ypi denotes the total population of the province, and coef signifies the national annual influenza attack rates. $\sum Y_{BISI}$ represents the total BISI search volume for the province, and ${BISI}_{Si}$ indicates the daily BISI search volume for the province.

The temporal span of the influenza season is determined using the MEM [27] to evaluate the start and end dates of the 2023–2024 influenza season across various provinces, employing the ILI+ index. Specifically, the epidemic onset in each province is identified as the first week when the ILI+ index exceeds the MEM-established threshold. The ILI+ index, which indicates the status of influenza infection, is derived by multiplying the ILI% by the influenza positivity rate [28,59]. This study assumed the earliest onset and latest conclusion dates estimated by MEM across different provinces as the influenza season in China and conducted modeling based on this time frame.

The BISI is utilized as a supplementary validation indicator. To minimize potential bias, we conducted multidimensional fitting of BISI and cross-validated it with the actual ILI+ data provided by the China CDC, drawing on recent methodology for influenza prediction in China [28].

This study accounts for the socioeconomic disparities among provinces by analyzing China’s 2023 per capita GDP data for each province. We employ k-means clustering and use the Euclidean distance to group the provinces. To determine the optimal number of clusters, the silhouette score is calculated for various cluster counts, with the clustering that achieved the highest silhouette score identified as the best clustering solution.

To estimate the influenza vaccination coverage rates for each province in 2023, we developed a Joinpoint regression model to analyze the trend in vaccination coverage from 2014 to 2022. This model enabled us to calculate the annual percentage change (APC), which quantifies the trend. We assumed that the change in vaccination coverage rates for 2023 would follow the pattern observed in the most recent trend. Using the 2022 data and the corresponding APC value, we projected the change in vaccination coverage rates for 2023. Consequently, we adopted the APC value from the final segment of the model estimation, which included 2022, as the trend change value for estimating the 2023 vaccination coverage rates. This value serves as a summary measure of recent trend changes over a fixed interval. Therefore, the influenza vaccination coverage rates for 2023 are calculated as follows:

(17) $$y_{2023}=y_{2022}*\left(1+APC*0.01\right)$$

where $y_{2023}$ represents the 2023 influenza vaccination coverage rates, and $y_{2022}$ represents the 2022 influenza vaccination coverage rates.

The model generates daily population dynamics for each compartment, and we employed calibration methods to estimate the transmission rate parameters. To ensure the validity and accuracy of the calibration, both the ILI+ index and BISI from various provinces are used to double-calibrate the model-simulated infection scale. Because the ILI+ index is a weekly metric, the model-simulated infection scale and BISI data are aggregated weekly for comparison. These three weekly standardized datasets are then max–min normalized, rescaling the largest data point to 1 and the smallest to 0. Subsequently, the correlation coefficients (*r*), root mean squared error (RMSE), and mean absolute error (MAE) are calculated between the normalized model-simulated infection scale and the ILI+ index and BISI to assess the calibration effectiveness. The transmission rate parameters with relatively high correlation coefficients and relatively low RMSE and MAE are selected as the optimal parameters.

The immunity acquired from previous epidemic seasons is indirectly reflected through the model calibration process. By calibrating the transmission rate β and estimating the vaccination coverage trend using the Joinpoint model, this approach accurately reflects the actual viral transmission intensity under current herd immunity conditions.

We used the optimal transmission rate parameter as our baseline scenario. Building on this foundation, we simulated various levels of increased vaccination coverage, considering multidimensional scenario differences, such as diverse vaccination timings, target populations, and geographic regions. The scenarios reflecting these dimensional variations are as follows.

(1) Regarding the timing of vaccination, we simulated three scenarios:
  (a) Achieving the vaccination target before the onset of the epidemic season.
  (b) Approximately 50% of the vaccination target is before the epidemic season, with the remaining vaccinations administered sequentially over the subsequent month.
  (c) Rushing to meet the vaccination target at the start of the epidemic season, followed by systematically completing the vaccination objectives within the next two months.
(2) Regarding the vaccinated population, we conducted simulations of the following scenarios:
  (a) Increasing vaccination coverage within a specific group.
  (b) Increasing vaccination coverage across any two age groups.
  (c) Increasing vaccination coverage for the entire population.
(3) Regarding the vaccinated regions, we conducted simulations of the following scenarios:
  (a) Increasing vaccination coverage within a single cluster of regions.
  (b) Increasing vaccination coverage across any cluster of regions.
  (c) Increasing vaccination coverage across all clusters of provinces on a national scale.

We evaluated the efficacy of different vaccination strategies by analyzing changes in vaccination coverage and disease burden. To simplify the model, we assumed that all populations received one dose of vaccine when calculating each indicator. Vaccination coverage was defined as the total number of vaccinated individuals divided by the total population. Disease burden metrics included incidence rates, severe case rates, and mortality rates derived from mathematical models established based on various influenza vaccination strategies.

The study constructed a Joinpoint regression model to calculate the Annual Percent Change (APC) of disease burden under different target vaccine coverage rates. The inflection point corresponding to the maximum APC that the model could identify as effectively reducing disease burden was defined as the immunization threshold. Beyond this threshold, even increasing vaccine coverage would still yield limited effects in reducing disease burden during influenza epidemic seasons. Based on the immunization threshold derived from the Joinpoint regression model, the vaccine coverage rates and disease burden reduction effects of different strategies at the immunization threshold can be determined by comparing them with the baseline scenario. We selected strategies with lower vaccine coverage rates that minimized disease burden as the optimal strategies.

The database was developed using Excel 2020 (Microsoft Corporation, Redmond, WA, USA). Statistical analyses, including MEM, and modeling, were conducted in Python 3.9 (Python Software Foundation, Beaverton, OR, USA), utilizing Jupyter Notebook version 6.4.12 (Project Jupyter, Austin, TX, USA). Trends analysis was performed using Joinpoint software version 4.9.1.0 (National Cancer Institute, Rockville, MD, USA). Statistical significance was established at a two-tailed significance level of *p* < 0.05.

## 3. Results

### 3.1. Estimation of Influenza Season, Vaccination Coverage, and Regional Clustering

**Figure 2:**
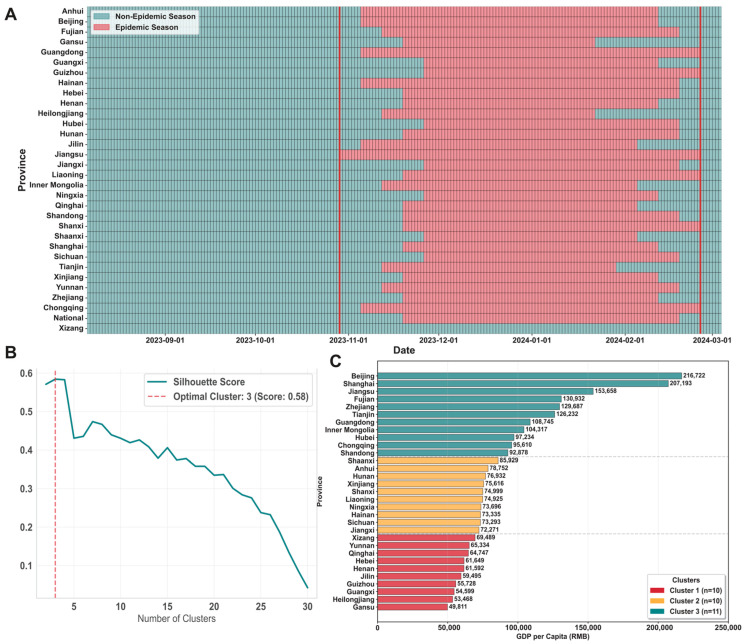
Estimation of influenza season and regional clustering. (**A**) Provincial epidemic seasons identified by the Epidemic Method (MEM). Vertical red lines mark the earliest provincial MEM-defined epidemic onset and the latest MEM-defined epidemic end used as the national simulation window. (**B**) Silhouette score used for cluster evaluation on the per capita GDP values of different numbers of clusters. The dashed vertical line indicates the selected optimum number of clusters. (**C**) Per capita GDP by province and cluster.

The MEM projects that the 2023–2024 influenza season for 31 provinces in China, excluding Xizang, which spans from 23 October 2023 to 25 February 2024 (Figure 2A). Xizang was excluded because its ILI+ index had only three weeks of valid surveillance data, making it impractical to estimate the start and end dates of the influenza season (Figure S1).

The Joinpoint model analysis revealed that from 2014 to 2022, influenza vaccination rates in most provinces followed an upward trend (APC > 0). In Beijing and Jiangsu Province, the rates initially decreased before rising (APC 1 < 0, APC 2 > 0), whereas in Xizang, they remained stable at first and then increased (APC 1 = 0, APC 2 > 0) (Figure S2). Trend forecasts suggest that the national vaccine coverage rate for the 2023–2024 influenza season is expected to be 2.89%. Tianjin is projected to have the highest coverage rate at 9.60%, whereas Hainan is anticipated to have the lowest at 0.58% (Figure S3).

Based on the 2023 per capita GDP data from various provinces, the optimal number of clusters, identified by the highest silhouette score, was three (Figure 2B). These clusters were classified as follows: underdeveloped regions (Cluster 1, consisting of 10 provinces), developing regions (Cluster 2, consisting of 10 provinces), and developed regions (Cluster 3, consisting of 11 provinces). Figure 2C displays the provinces included in each cluster along with their respective per capita GDP.

### 3.2. Spatiotemporal Differences Across Various Clustered Regions

**Figure 3:**
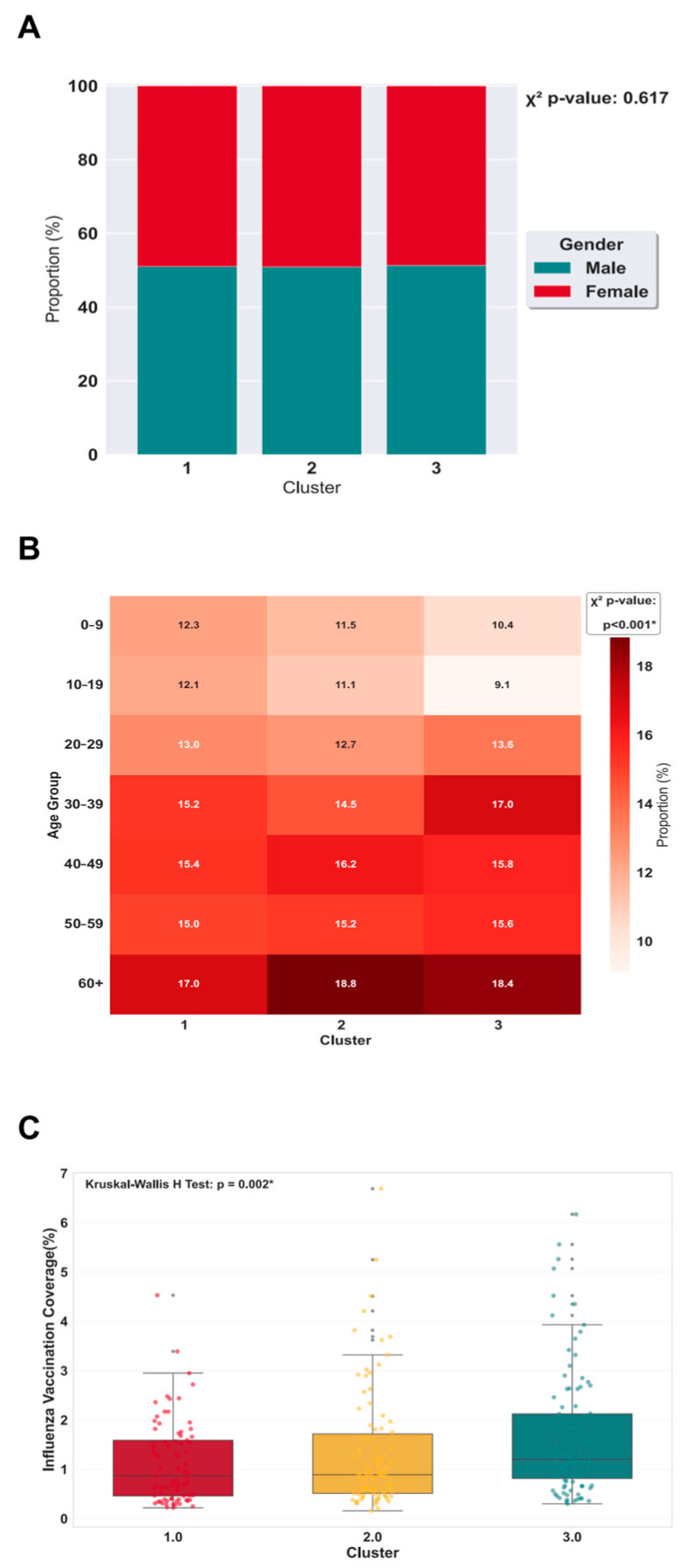
Differences across socioeconomic clusters by sex, age, and influenza vaccine coverage. (**A**) Sex composition by cluster (**B**) Age-structure heatmap by cluster (**C**) Influenza vaccine coverage by cluster. Boxplots summarize province-level influenza vaccination coverage (%) within each cluster. ‘*’ denotes a statistically significant difference (*p* < 0.05).

In terms of demographic structure, there were no significant differences in gender ratios between different cluster regions (*p* = 0.617) (Figure 3A). Regarding age distribution, developing regions (Cluster 2) had the highest proportion of ≥65 years age groups (17.0%); underdeveloped regions (Cluster 1) exhibited higher percentages of middle-aged groups (40–49 years, 16.2%) and elderly populations (18.8%); whereas developed regions (Cluster 3) were characterized by a higher proportion of 30–39 year groups (17.0%) and elderly individuals (18.4%). The intergroup differences were statistically significant (χ^2^ test, *p* < 0.001) (Figure 3B). Additionally, Figure 3C demonstrated that influenza vaccine coverage rates across the three regions ranked from highest to lowest as developed regions, developing regions, and underdeveloped regions, with statistically significant intergroup differences (Kruskal–Wallis test, *p* < 0.001).

**Figure 4:**
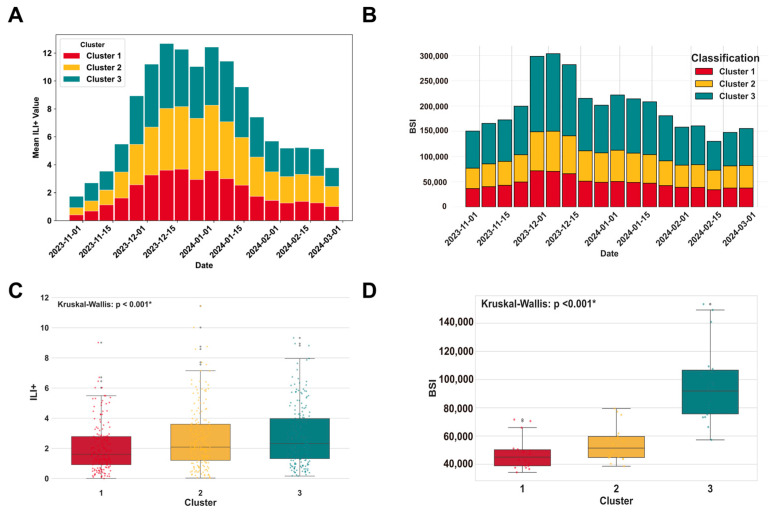
Differences in ILI+ (influenza-like illness positive) index and Baidu Influenza Search Index (BISI) across socioeconomic clusters. (**A**) Epidemic curves of the mean weekly ILI+ value by cluster. (**B**) Time series curve of the weekly BISI by cluster. (**C**) Distribution of weekly ILI+ by cluster. (**D**) Distribution of weekly BISI by cluster. ‘*’ denotes a statistically significant difference (*p* < 0.05).

The ILI+ index and BISI in different clustering regions exhibited a trend of first increasing and then decreasing (Figure 4A,B). In developed regions (Cluster 3), the peak values of both indicators appeared earliest in mid-December 2023, with BISI peaking at approximately 310,000 and ILI+ index peaking at around 12–13, showing the greatest overall fluctuation amplitude. In developing regions (Cluster 2), the peak time lagged by 1–2 weeks, with BISI peaking at approximately 180,000 and ILI+ index peaking at moderate levels. In underdeveloped regions (Cluster 1), both indicators reached their lowest peaks (BISI at approximately 70,000 and ILI+ index at 3–4), appeared the latest (after late December 2023), and remained at relatively high levels until February 2024, exhibiting a longer epidemic cycle. The inter-regional differences between the ILI+ index and BISI were statistically significant (Kruskal–Wallis test, all *p* < 0.001) (Figure 4C,D).

### 3.3. Model Simulation and Validation

**Figure 5:**
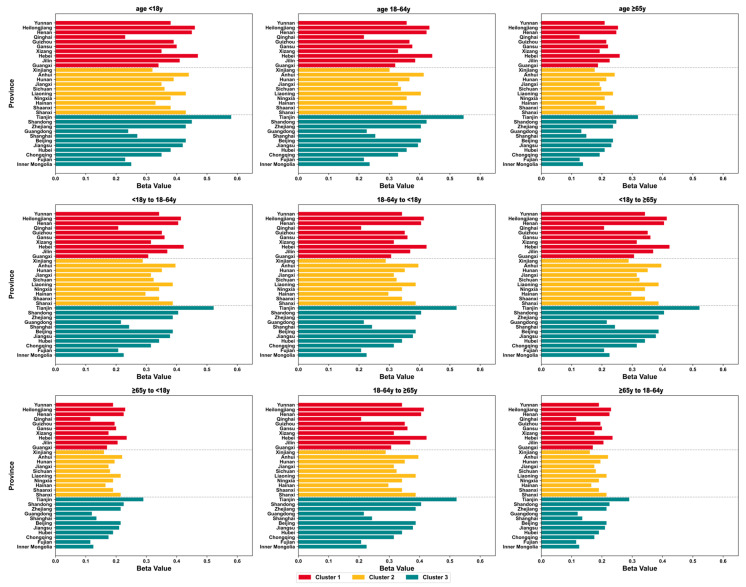
Regional and age-related differences in influenza relative transmission rate values.

Model simulation results revealed significant regional and age-related differences in influenza transmission Beta values: Developed regions (Cluster 3) exhibited the highest transmission Beta values across all age groups and inter-age groups, particularly in the 18–64 age group and the cross-age transmission pathways between 18 and 64 years and <18 years/≥65 years. Developing regions (Cluster 2) showed intermediate Beta values, indicating moderate transmission intensity. Underdeveloped regions (Cluster 1) demonstrated the lowest Beta values across all pathways, suggesting weaker population mobility and social clustering, with relatively limited influenza transmission potential. The 18–64 age group remained the core transmission vector across all regions, exhibiting significantly higher bidirectional transmission Beta values with <18-year-old minors and ≥65-year-old elderly populations compared to other inter-age pathways (Figure 5).

**Figure 6:**
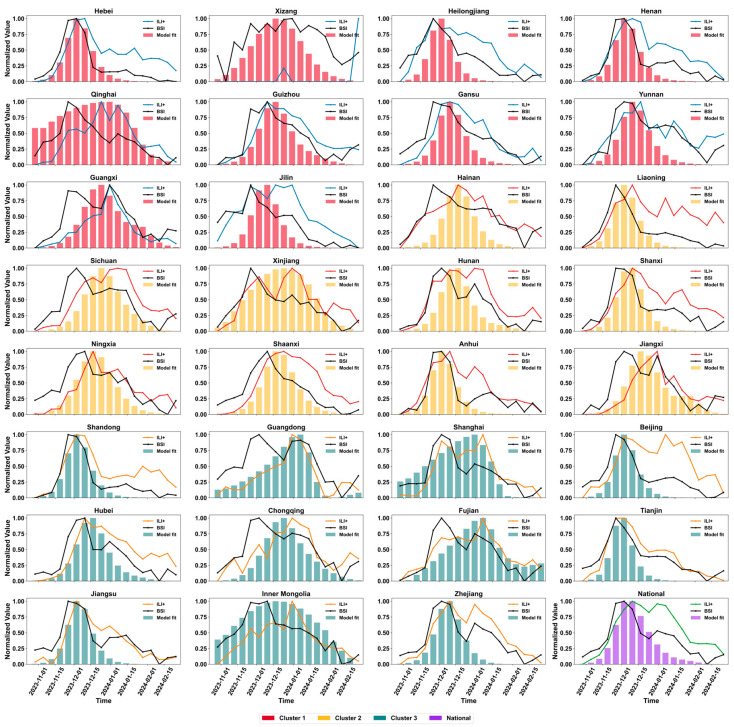
The model-simulated epidemic validated with observed influenza case numbers, ILI+, and BISI trend. ILI+, influenza-like illness positive; BISI, Baidu Influenza Search Index.

**Figure 7:**
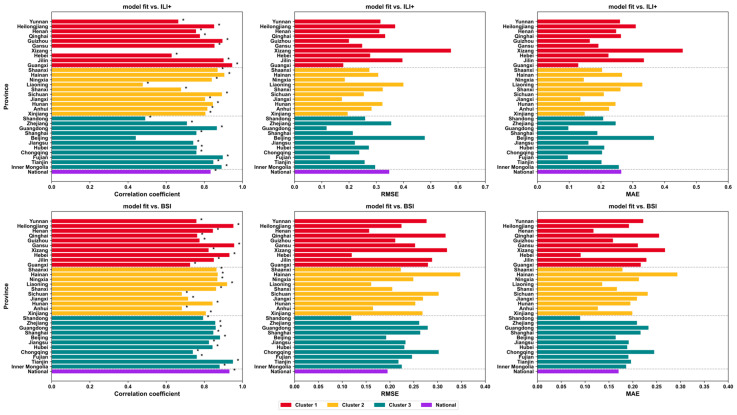
The model-simulated values of ILI+ index and BISI with observed data. ILI+, influenza-like illness positive; BISI, Baidu Influenza Search Index. ‘*’ denotes a statistically significant difference (*p* < 0.05).

The epidemic transmission trajectories simulated by the model showed high consistency with the observed influenza case numbers, ILI+, and BISI trends in most provinces (Figure 6). The normality test results for influenza case numbers, ILI+ index, and BISI demonstrated that all three indicators showed *p* < 0.001 significance at both the provincial and national levels (Figures S4–S6), indicating that ILI+, simulated case numbers, and BISI do not conform to normal distribution. Both the simulated values of ILI+ index and BISI exhibited significant correlations with actual observed values (Figure 7). Regional variations were observed in the simulation performance of ILI+ index and BISI: developed regions (Cluster 3) demonstrated optimal simulation accuracy, with ILI+ index simulation MAE mostly ranging from 0.1 to 0.2; BISI simulation MAE was mostly below 0.20 and RMSE was between 0.10 and 0.30. Developing regions (Cluster 2) exhibited moderate simulation accuracy, with ILI+ index simulation MAE ranging from 0.2 to 0.4 and RMSE from 0.2 to 0.5, while BISI simulation MAE ranged from 0.15 to 0.25 and RMSE from 0.15 to 0.30. Underdeveloped regions (Cluster 1) showed slightly higher simulation errors, with ILI+ index simulation MAE ranging from 0.3 to 0.5, RMSE from 0.3 to 0.6, BISI simulation MAE from 0.20 to 0.30, and RMSE from 0.20 to 0.35 (Figure 7). The national-level simulation performance was comparable to that of developed regions, with correlation coefficients between ILI+ index and BISI both exceeding 0.7, collectively validating the model’s strong ability to replicate influenza epidemic trends and disparities across regions with varying economic development levels.

**Figure 8:**
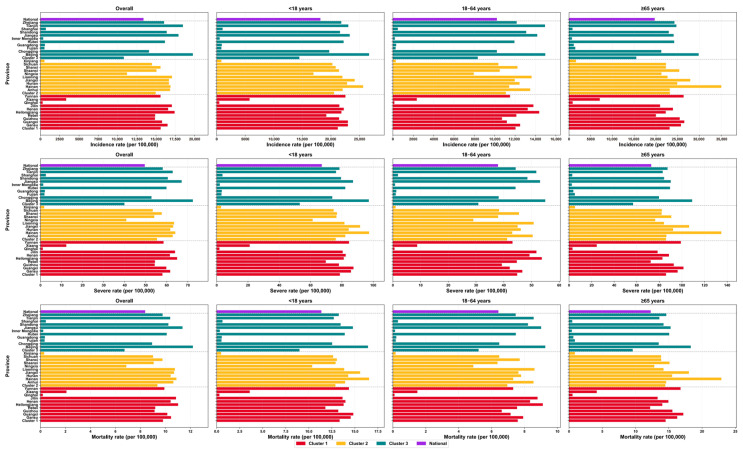
The model-simulated disease burden in the baseline scenario.

Using the optimal transmission rate parameter as the baseline scenario, the disease burden simulation results (Figure 8) indicate that the baseline incidence rate, severity rate, and mortality rate were 13,374.93 cases, 49.52 cases, and 8.37 cases per 100,000 population, respectively. Developed regions (Cluster 3) exhibited the highest disease burden, with an overall incidence rate of approximately 15,000–20,000 cases per 100,000 population, while both the severe case rate and mortality rate were the highest among the three regions. Developing regions (Cluster 2) followed closely, while the underdeveloped regions (Cluster 1) had the lowest overall incidence rate of around 5000–10,000 cases per 100,000 population. The disease burden across all three regions demonstrated age-related patterns, with the 18–64-year-old young and middle-aged population showing relatively lower incidence rates, severe case rates, and mortality rates. In contrast, the elderly population aged ≥ 65 years bore a heavier disease burden, with the highest incidence rate reaching up to 35,000 cases per 100,000 population.

### 3.4. Simulation of Optimal Influenza Vaccination Strategies

**Figure 9:**
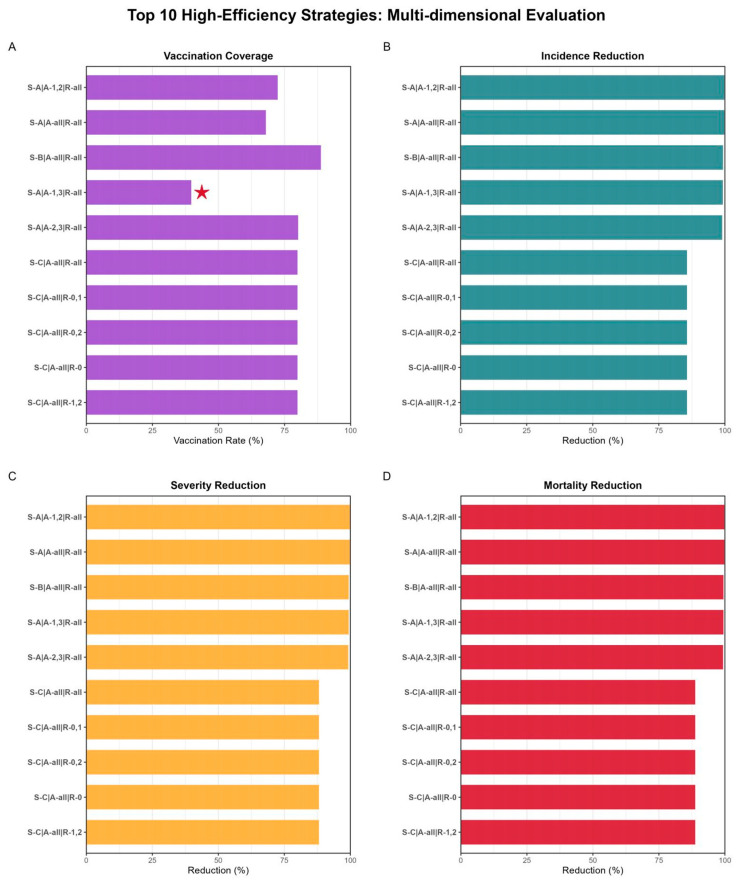
Simulation results of optimal vaccination strategies. (**A**) Threshold effect diagram of vaccination coverage rate, red star denotes the optimal vaccination strategy; (**B**) threshold effect plot of incidence rate decline percentage; (**C**) threshold effect plot of severity rate decline percentage; (**D**) threshold effect plot of mortality rate decline percentage.

Comprehensively considering factors such as vaccination timing, target populations, and geographic regions, the model simulated disease burden under 147 strategies with varying target vaccination coverage rates (percentage increase from baseline levels) ranging from 0 to 100. The Joinpoint regression model analysis revealed that the threshold ranges for all 147 strategies were 50–97% (Figure S7, Table S1). Most strategies could achieve immunization thresholds at a 74% increase in vaccination coverage compared to baseline levels. Beyond this threshold, further increases in vaccination coverage would not significantly enhance disease burden reduction effects. Based on the immunization threshold results derived from vaccination strategies, we calculated the vaccination rates and disease burden reduction effects (including percentage decreases in incidence rate, severe case rate, and mortality rate) for each strategy at the threshold state (Figures S8–S11) and identified the optimal vaccination strategy. The simulation results of optimal vaccination strategies (Figure 9) demonstrated that the optimal strategy involves increasing influenza vaccine coverage rates by 93% above baseline levels for both the under-18 age group and ≥65-year-old population across all provincial clusters nationwide before the onset of the epidemic season (covering 39.73% of the national population). This strategy meets the threshold requirements for vaccination coverage while achieving optimal disease burden reduction effects, resulting in significant decreases of 99.26%, 99.42%, and 99.46% in incidence rate, severe case rate, and mortality rate, respectively. Prior to the onset of the epidemic season, achieving a vaccination coverage rate 62% higher than baseline levels across the entire population (covering 67.92% of the national population) and a vaccination coverage rate 79% higher than baseline levels for the under-18 age group and the 18–64 age group (covering 72.39% of the national population) could yield disease burden reduction effects comparable to optimal strategies. Furthermore, within two months after the outbreak, increasing vaccination coverage to threshold levels either for the entire population or for underdeveloped regions (Cluster 1) (either independently or in combination with any other region) could reduce incidence rates by 85.70%, severe case rates by 88.75%, and mortality rates by 88.77%, respectively. In contrast, simulations of non-optimal strategies targeting the highly active 18–64 age group revealed significantly lower efficiency. For instance, prior to the epidemic season, even with an immunity threshold as high as 95% for the 18–64 age group alone, the reductions in incidence, severe cases, and mortality rates were only 52.27%, 59.36%, and 60.89%, respectively. Although combined strategies involving the 18–64 age group and either the under-18 group (threshold 79%, reduction >99%) or the ≥65 group (threshold 97%, reduction >98%) could achieve substantial protection, these scenarios require covering over 70% of the national population—a much larger proportion compared to the 39.73% required by the optimal strategy recommended in this study.

The strategy names in the diagram illustrate the combination schemes of different strategies: S stands for “sequence strategy,” which refers to administering vaccinations in chronological order. Specifically, S denotes sequence, representing sequential vaccination strategies: S-A indicates achieving vaccination targets before the start of the epidemic season; S-B indicates that approximately 50% of the target vaccination population must be vaccinated before the epidemic season, with the remaining vaccinations conducted sequentially within the following month; S-C indicates achieving full vaccination targets during the initial outbreak phase, followed by systematic completion of the vaccination plan over the next two months. A denotes age, representing age-specific strategies: 1, 2, and 3 represent populations under 18 years old, 18–64 years old, and 64 years and above, respectively; A-1, A-2, and A-3 indicate increased vaccination coverage within specific age groups; A-1,2, A-1,3, and A-2,3 indicate increased vaccination rates for two specific age groups; A-all denotes vaccination coverage across all age groups. R denotes region, representing regional strategies: 0, 1, and 2 indicate underdeveloped regions, developing regions, and developed regions, respectively; R-0, R-1, and R-2 indicate increased vaccination coverage within specific regions; R-0,1, R-0,2, R-1,2 indicate increased vaccination rates for two specific age groups; R-all denotes vaccination coverage across all regions. 

## 4. Discussion

Our model establishes a multidimensional transmission dynamics framework, categorized by province and age group, incorporating the impact of actual population movements. It undergoes validation against ILI+ and BISI data using correlation coefficients to assess robustness, enabling the evaluation of influenza epidemic burden under various vaccination strategies. The China Center for Disease Control and Prevention releases the China Influenza Vaccination Technical Guidelines annually, but existing strategies have not fully considered the impact of regional socioeconomic disparities on vaccine distribution. To address the public health practice need of reducing influenza burden, it is urgently necessary to conduct model studies on regional vaccination strategies by integrating differentiated transmission characteristics across regions. This study employs a transmission dynamics model incorporating population mobility characteristics to simulate influenza epidemic trends from 2023 to 2024. It focuses on evaluating disease burden changes under differentiated vaccination strategies across regions, quantitatively analyzing the differential impacts of region-specific vaccination coverage targets on incidence rates, severe case rates, and mortality rates. The study identifies a 93% vaccination coverage rate among key populations as a critical prevention threshold, providing scientific evidence for developing region-specific precision influenza vaccination strategies and facilitating optimal disease burden control at the national level.

The study results indicate that developed regions (Cluster 3), characterized by high population density, intensive social contact, and frequent population mobility (manifested as the highest transmission Beta values), exhibit epidemic features of “high intensity, early peak onset, and short cycle duration.” The ILI+ index and BISI peak values in these regions are significantly higher than those in other areas, posing severe challenges to acute medical capacity in local hospitals. The epidemiological characteristics observed in this region exhibit high consistency with real-world influenza transmission patterns. Urbanization levels, population density, and cross-regional mobility have been demonstrated to significantly enhance influenza transmission intensity and epidemic peak levels [60]. The transmission dynamics model based on population mobility networks can also effectively replicate the spatiotemporal diffusion patterns of influenza across different regions [61], indicating that this model possesses reliable biological validity in simulating regional epidemic variations. The model simulation and validation results further confirm the regional heterogeneity of influenza epidemics. The Beta values for transmission within and across age groups were the highest in developed regions. The 18–64-year-old young and middle-aged population, due to extensive social activities and high contact frequency, became the key transmission hub for influenza across age groups [62]. This finding aligns with research conclusions on household and community transmission, which also explains why developed regions, despite possessing abundant medical resources, still ranked first in disease burden metrics (incidence rate, severe case rate, and mortality rate). In contrast, less developed regions (Cluster 1), despite exhibiting “low-intensity and delayed onset peaks” characteristics due to lower mobility, demonstrated longer epidemic cycles and higher proportions of middle-aged and elderly populations, indicating persistent social transmission pressure and significantly elevated risk of severe outcomes among susceptible groups. It is noteworthy that during model validation, significant differences in simulation accuracy were observed across regions. The simulation errors in underdeveloped areas were notably higher than those in developed and developing regions, with both the MAE and RMSE of ILI+ index and BISI simulations being the highest among the three categories. This disparity is attributed to multiple factors: Firstly, the construction of public health surveillance systems in underdeveloped areas lags behind, with some regions represented by Xizang only providing limited effective surveillance data, resulting in suboptimal monitoring quality and data integrity, which directly impacts the accuracy of model calibration. Secondly, although population mobility is generally low in underdeveloped areas, the dispersed population distribution and challenges in grassroots data collection lead to insufficient alignment between population mobility and epidemiological surveillance data. Thirdly, vaccine coverage rates in underdeveloped areas are significantly lower, and regional disparities in population immunity levels further complicate the model’s ability to fit disease transmission trends.

Based on the optimal strategy derived from model results, it is recommended to increase vaccination coverage rates by 93% (covering approximately 40% of the national population) among key populations under 18 years of age and over 65 years of age before the epidemic season, which could achieve substantial benefits of reducing disease burden by more than 99%. Although young and middle-aged adults (18–64 years old) play a pivotal role in influenza transmission, this study prioritizes increasing vaccination rates among individuals under 18 years of age and those over 65 years of age. The rationale for this decision is twofold: firstly, these age groups are at high risk of severe illness and mortality, and directly protecting them can significantly alleviate pressure on healthcare systems; quantitatively, our results show that vaccinating the 18–64 age group alone, even at a 95% immunity threshold, only reduces the disease burden by 52.27%~60.89%, whereas the optimal strategy achieves superior protection by covering only 39.73% of the population. Secondly, schools, as high-density venues for minors, serve as the primary source for the transition of influenza from clustered outbreaks to community transmission, and targeted establishment of an immune barrier can yield substantial indirect protective effects. Furthermore, compared to the 18–64 age group who often face “time poverty” and logistical barriers as the primary workforce, children and the elderly can be more effectively reached through school-based and community-based health management systems, which offer higher operational feasibility and compliance. This strategy precisely targets critical nodes in the influenza transmission chain, effectively protecting high-risk populations while simultaneously interrupting transmission routes spreading from schools and households to the broader community. However, there remains a significant gap between the currently predicted national vaccination rate (2.89%) and this immunization threshold, a disparity closely related to the regional disparities in influenza vaccination coverage observed in China.

Domestic studies indicate significant spatiotemporal and regional disparities in influenza vaccination coverage. Since Beijing pioneered free vaccination for the elderly and students in 2007, many regions have gradually followed suit, but there are substantial differences in policy coverage and subsidy levels [63]. In regions with free vaccination programs such as Beijing and Zhejiang, the vaccination rate among the elderly ranges from 20% to 53%, whereas provinces where vaccination is primarily self-paid generally report rates below 5% [64]. The vaccination pattern characterized by “high rates in eastern regions, low rates in western regions, superior performance in urban areas, and weaker outcomes in rural areas” is closely associated with fiscal investment, healthcare security models, and vaccination accessibility. Notably, the low vaccination rate among elderly populations in rural areas remains particularly prominent [64], further widening the gap compared to the theoretically optimal vaccination level. Compared with the domestic situation, the influenza vaccination coverage and safeguarding models in developed countries as well as Hong Kong, Macao, and Taiwan regions are more mature, exhibiting a significant gap compared to China: the vaccination rate among elderly individuals in the UK ranges from 70% to 78% [65], while in South Korea it is approximately 80% [66]. The vaccination rate reached 51.5% for people aged 65 and above in Hong Kong, China, and 73.8% for children aged 6–11, achieved through government funding, school outreach, and community vaccination networks to ensure high coverage [67]. Canada targets an 85% vaccination rate among high-risk groups [68], the WHO advocates for a 75% rate within this demographic [69], and the United States aims for a 70% rate across the entire population [70]. Taiwan, China, has a flu vaccination rate of 44.8% among those aged 65 and above, significantly higher than the mainland level, mainly due to the full coverage of universal health insurance and the widespread medical service network [71]. It is noteworthy that post-COVID-19, vaccination behaviors exhibited a bidirectional effect: on the one hand, the pandemic heightened public awareness of respiratory infectious disease prevention, objectively creating favorable conditions for influenza vaccine promotion; on the other hand, the global spread of vaccine hesitancy led to public concerns about vaccine safety and necessity extending from COVID-19 vaccines to influenza vaccines [72], posing new challenges to the improvement of influenza vaccination rates in China. In summary, the overall influenza vaccination rate in China remains low with significant regional disparities, showing considerable gaps compared to developed countries and regions. Vaccine hesitancy and immunization fatigue in the post-COVID era have further exacerbated the challenges in improving vaccination rates [73]. Theoretically, a high vaccination rate target of 93% can effectively establish herd immunity. However, in practice, it faces multiple practical barriers such as economic costs, service accessibility, public awareness, and vaccine trust, making direct implementation challenging [74]. For example, digital messaging can effectively enhance vaccination willingness among China populations [75], while localized interventions such as “pay-it-forward” have demonstrated significant effects in children and elderly populations, with costs lower than traditional out-of-pocket models, providing evidence-based support for cost-effective expansion of coverage [76].

Through a comprehensive analysis of successful experiences from developed countries and China’s Hong Kong, Macao, and Taiwan regions, their core strategies can be distilled into three common characteristics, all of which exhibit strong adaptability to China’s local context and scalability: first, government-led fiscal safeguards, achieving low-cost or free vaccination through special government funding and universal health insurance coordination, thereby eliminating vaccination barriers at the economic root level; second, building comprehensive vaccination accessibility by establishing a collaborative vaccination network involving schools, communities, and primary healthcare institutions, tailored to different population lifestyles to reduce vaccination behavioral costs; third, targeted promotion for high-risk groups such as the elderly and children through specialized outreach programs, enhancing prevention efficiency and resource utilization precision. Although the model-derived coverage rate of 93% for the key population (approximately 40% of the total population) shows a significant gap compared to China’s current vaccination rate of around 3%, this figure should be regarded as an idealized theoretical immune threshold rather than a mandatory administrative target in the short term. In public health practice, achieving this goal requires a stepwise approach. Based on China’s national conditions, regional development disparities, and the aforementioned experiences, targeted implementation recommendations are proposed as follows: First, leveraging the tiered fiscal system, gradually include high-risk populations under 18 years old and aged ≥ 65 years in the fiscal coverage scope for free vaccination, promoting the extension of free vaccination policies from developed eastern regions to underdeveloped western regions and from urban to rural areas to narrow regional policy gaps. Second, drawing on the community vaccination network models of Hong Kong, Macao, and Taiwan, improve the layout of vaccination sites at community health service centers in urban areas and establish grassroots vaccination service systems in rural areas through township health centers and village clinics to address insufficient vaccination accessibility in remote regions. Third, integrating digital communication and localized intervention experiences, disseminate influenza prevention and vaccine safety knowledge through new media platforms, conduct targeted publicity campaigns in schools, communities, and rural areas to alleviate vaccine hesitancy and enhance public vaccination willingness. Fourth, fully utilize China’s unique counterpart support mechanism [77,78] to facilitate targeted assistance from developed provinces to underdeveloped regions in areas such as influenza surveillance capacity building, vaccination service network establishment, grassroots disease prevention and control talent training, and emergency drug reserves, extending from simple resource support to comprehensive capacity building to effectively reduce regional disparities in public health services. Fifth, set tiered vaccination targets by region: developed areas should first benchmark against developed countries and Hong Kong, Macao, and Taiwan levels to achieve vaccination rates exceeding 50% for high-risk populations, while underdeveloped areas should prioritize eliminating vaccination service gaps and achieving basic coverage for high-risk populations, steadily advancing toward the national vaccination target of 40% of the total population. Through a comprehensive strategy featuring government leadership, fiscal support, regional coordination, and scenario-based implementation, we aim to address the core challenges of low influenza vaccination rates and significant regional disparities in China, thereby steadily advancing vaccination coverage toward the theoretically optimal threshold.

The multidimensional transmission dynamics model constructed in this study, incorporating population mobility characteristics, provides a new perspective for understanding the heterogeneity of influenza transmission across different regions of China. The identified regional epidemic disparities reveal the limitations of a unified vaccination strategy. The simulation-derived targets of 93% vaccine coverage for key populations and the benefits of reduced disease burden offer clear quantitative evidence for policy-making and public health resource allocation, as well as a reference for regions with similar developmental disparities. This study has significant advantages, including the incorporation of regional economic gradients, interactions between population mobility and age structure, and quantification of epidemic heterogeneity across regions with varying development levels, thereby providing data support for differentiated prevention and control measures. However, it still has certain limitations: first, the baseline vaccine coverage rate used in the model is extrapolated from historical trends rather than actual vaccination rate data monitored in each province, which may lead to deviations between baseline parameters and real-world scenarios; second, this study only analyzed a single influenza epidemic season and did not incorporate dynamic changes in viral strain mutations, population immunity accumulation, and vaccine efficacy across multiple consecutive epidemic seasons, making it difficult to reflect long-term epidemiological patterns; third, this study did not conduct cost-effectiveness or other health economics analyses, thus failing to provide more actionable decision-making references for resource-limited regions; fourth, the model uniformly assumed one dose per person, which differs from China’s current influenza vaccination technical guidelines—children aged 6 months to 8 years require two doses of inactivated vaccines with a minimum interval of 4 weeks for the first vaccination, while individuals aged 9 years and above need only one dose of either inactivated or live attenuated vaccines [79]. This simplified assumption may underestimate the complexity of vaccination schedules and practical implementation challenges for younger children. Future research may incorporate long-term surveillance data across multiple epidemic seasons and regional vaccination rates to optimize baseline parameters, integrate a health economics evaluation framework, and strictly adhere to national influenza vaccination technical guidelines for distinguishing age groups, vaccine types, and dosing schedules. Additionally, incorporating more influencing factors to refine model structures will provide more comprehensive and practical decision-making support for precision and differentiated influenza prevention and control strategies.

## 5. Conclusions

In conclusion, the findings of this study indicate significant heterogeneity among regions with different levels of economic development in China regarding influenza epidemic patterns, transmission intensity, and disease burden. A uniform vaccination strategy fails to accommodate regional disparities. Policymakers should implement differentiated and targeted interventions based on regional economic gradients, promote equitable vaccine resource allocation through paired assistance from developed to underdeveloped regions, prioritize vaccination for high-risk populations aged 18 years and above and 65 years and above, and achieve the goal of vaccinating 40% of the total national population. This approach will minimize influenza incidence rates, severe case rates, and mortality rates while enhancing the overall prevention and control efficiency and equity.

## Figures and Tables

**Table 1:** Descriptive information of parameters in the model.

Parameter	Definition	Unit	Value	Range	Source
$\beta_{i},\beta_{j},\beta_{ij},\beta_{ji}$	Transmission rates of human-to-human within or between age groups.	-		≥0	Calibration
*k*	Relative transmissibility coefficient of asymptomatic to symptomatic individuals		0.500	0.125–0.500	[29,30,31,32,33,34]
*p*	Proportion of the asymptomatic infections	%	0.333	0.010–0.820	[29,31,32,34,35,36,37,38,39,40,41,42]
*q*	Probability of symptomatic progression to severe disease	%	0.003	0.003–0.005	[43,44]
*f*	Case fatality ratio among critically infected individuals	%	0.170	0.050–0.650	[43,44,45,46,47,48,49]
$\omega_{S}$	Incubation relative transition rate	days-1	0.53	0.140–1.000	[29,30,33,50]
$\omega_{A}$	Latent relative transition rate	days-1	0.83	0.140–1.000	[29,30,33,50]
$\gamma_{A}$	Recovery rate for asymptomatic individuals	days-1	0.25	0.070–1.000	[29]
$\gamma_{s}$	Recovery rate for symptomatic individuals	days-1	0.31	0.071–1.000	[30,33,50]
$n_{ij}$	Vaccine coverage rate by age group	%		0–1	Real-data
$V{ES}_{ij}$	VE reducing susceptibility	Age <18	0.65	0.06–0.97	[35,51,52,53,54,55]
		Age 18–64	0.50	0.01–0.89	
		Age ≥65	0.42	0.14–0.80	
$V{EI}_{ij}$	VE reducing infectiousness	-	0.80	0–1	[35]
$V{EC}_{ij}$	VE reducing critical severity	Age <18	0.69	0.16–0.98	[20,56]
		Age 18–64	0.49	0.05–0.74	
		Age ≥65	0.50	0.05–0.88	
$V{EF}_{ij}$	VE reducing mortality	-	0.29	0.19–0.33	[57]
θ	Vaccination rate of transitions from *S* to *V*		-	0–1	Assumed

## Data Availability

The data presented in this study are available on request from the corresponding author due to the national legal regulations and internal policy.

## Supplementary Materials

The following supporting information can be downloaded at: https://www.mdpi.com/article/10.3390/vaccines14050425/s1, Table S1. Summary table of different strategy threshold range. Figure S1. ILI+ scatter diagram: the horizontal axis represents dates, and the vertical axis represents the ILI+ (positive for influenza-like symptoms) value. Figure S2. Annual Percent Change: the horizontal axis represents years, and the vertical axis represents influenza vaccine coverage rates. The curves in the figure illustrate annual percentage change trends. When APC > 0, it indicates an increasing trend in influenza vaccination rates, while APC < 0 suggests a declining trend in influenza vaccination rates. Figure S3. Vaccination coverage rates by province: the horizontal axis represents influenza vaccination coverage rate, and the vertical axis represents provinces. Figure S4. Simulation of normal distribution for influenza cases: the horizontal axis represents the number of influenza cases, while the vertical axis indicates sample frequency. Each plot depicts conditions across different provinces or nationwide. Distinct colors denote regional variations: red, yellow, and green correspond to Cluster 1, Cluster 2, and Cluster 3 respectively, with purple representing the national overall level. All subplots underwent normality testing, with results consistently showing *p* < 0.001. Figure S5. Simulation of normal distribution for positive index of influenza symptoms: the horizontal axis represents the influenza symptom positivity index, while the vertical axis indicates sample frequency. Each chart displays data distribution across different provinces or nationwide. Regional variations are represented by distinct colors: red, yellow, and green correspond to Cluster 1, Cluster 2, and Cluster 3 respectively, with purple indicating the national overall level. All subplots underwent normality testing, with consistent results showing *p* < 0.001. Figure S6. Normal distribution simulation of Baidu Influenza Search Index: the horizontal axis represents the Baidu Influenza Search Index, while the vertical axis indicates sample frequency. Each chart displays data conditions across different provinces or nationwide. Distinct colors represent regional variations: red, yellow, and green correspond to Cluster 1, Cluster 2, and Cluster 3 respectively, with purple indicating the national overall level. All subplots underwent normality testing, with consistent results showing *p* < 0.001. Figure S7. Forest plot of different strategy threshold values: the horizontal axis represents percentages, and the vertical axis shows different strategy names. Different colors indicate vaccination rates, incidence rates, severe case rates, and mortality rates. This figure displays the threshold values and error ranges for each strategy derived from Joinpoint regression analysis results. Figure S8. Threshold effect diagram of vaccination coverage rate: the horizontal axis represents vaccination rates, while the vertical axis indicates different strategies. Strategies marked with five-point stars represent the best selected strategies. Figure S9. Threshold effect plot of incidence rate decline percentage: threshold effect plot of percentage reduction in incidence rate: the *x*-axis represents the percentage reduction in incidence rate, and the *y*-axis represents different strategies. Figure S10. Threshold effect plot of severity rate decline percentage: threshold effect plot of percentage reduction in severity rate: the *x*-axis represents the percentage reduction in incidence rate, and the *y*-axis represents different strategies. Figure S11. Threshold effect plot of mortality rate decline percentage: threshold effect plot of percentage reduction in mortality rate: the *x*-axis represents the percentage reduction in incidence rate, and the *y*-axis represents different strategies.

## Abbreviations

ARIs	acute respiratory infections
COVID-19	coronavirus disease 2019
HIV	human immunodeficiency virus
RSV	respiratory syncytial virus
China CDC	Chinese Center for Disease Control and Prevention
ILI-pr	influenza-like illness positivity rates
ILI%	influenza-like illness proportions
BISI	Baidu Influenza Search Index
CBMI	China Baidu Migration Index
AAPC	Average Annual Percent Change
SVEAICFR model	Susceptible-Vaccinated-Exposed-Asymptomatic-Infectious-Critical-Fatal-Recovered/Removed model
ILI+	Influenza-Like Illness Positive Index
r	correlation coefficient
MSE	mean squared error
RMSE	root mean squared error
SD	standard deviation
IQR	interquartile range
NNDSS	the National Notifiable Disease Surveillance System
PIV-3	parainfluenza virus type 3
MTB	Mycobacterium tuberculosis
