# Supplementary material for: A Human Mobility-Based Modeling Study of Influenza Vaccination Strategies Across Socioeconomically Disparate Regions in China
Source: Vaccines (Basel). 2026 May 9;14(5):425. doi: 10.3390/vaccines14050425 (PMC13211672; doi:10.3390/vaccines14050425)
Supplement: Supplementary file 1 [file vaccines-14-00425-s001.zip › vaccines-4280095-supplementary.pdf]

Table S1. Summary Table of Different Strategy Threshold Range

vt	Age strategy	Regional Strategy	Incidence threshold	Severity threshold	Mortality threshold	Range of threshold
10	all_groups	all_clusters	63 (62, 65)	62 (61, 64)	62 (61, 64)	62% - 63%
10	all_groups	cluster0_1	52 (51, 53)	52 (50, 53)	51 (50, 52)	51% - 52%
10	all_groups	cluster0_2	50 (49, 52)	55 (49, 58)	54 (51, 58)	50% - 55%
10	all_groups	cluster0_only	55 (49, 59)	53 (52, 57)	54 (51, 57)	53% - 55%
10	all_groups	cluster1_2	51 (50, 52)	50 (49, 57)	50 (49, 58)	50% - 51%
10	all_groups	cluster1_only	52 (50, 53)	51 (50, 52)	51 (50, 52)	51% - 52%
10	all_groups	cluster2_only	51 (49, 56)	50 (49, 54)	51 (49, 52)	50% - 51%
10	group1_2	all_clusters	80 (78, 81)	79 (78, 80)	79 (78, 81)	79% - 80%
10	group1_2	cluster0_1	67 (66, 68)	66 (65, 67)	66 (65, 67)	66% - 67%
10	group1_2	cluster0_2	64 (63, 65)	63 (62, 71)	68 (62, 73)	63% - 68%
10	group1_2	cluster0_only	64 (63, 66)	69 (66, 73)	69 (66, 72)	64% - 69%
10	group1_2	cluster1_2	66 (65, 67)	65 (64, 66)	65 (64, 66)	65% - 66%
10	group1_2	cluster1_only	67 (65, 68)	66 (65, 67)	66 (65, 67)	66% - 67%
10	group1_2	cluster2_only	65 (62, 69)	64 (61, 66)	64 (61, 66)	64% - 65%
10	group1_3	all_clusters	94 (92, 96)	93 (91, 95)	93 (91, 95)	93% - 94%
10	group1_3	cluster0_1	82 (80, 83)	80 (79, 82)	80 (79, 81)	80% - 82%
10	group1_3	cluster0_2	80 (79, 82)	79 (78, 80)	79 (78, 80)	79% - 80%
10	group1_3	cluster0_only	80 (79, 81)	79 (78, 80)	79 (77, 80)	79% - 80%
10	group1_3	cluster1_2	80 (78, 81)	78 (77, 80)	78 (77, 79)	78% - 80%
10	group1_3	cluster1_only	79 (77, 80)	77 (76, 79)	77 (76, 79)	77% - 79%
10	group1_3	cluster2_only	75 (74, 77)	74 (73, 75)	74 (72, 85)	74% - 75%
10	group1_only	all_clusters	96 (94, 98)	96 (94, 98)	96 (94, 98)	96% - 96%
10	group1_only	cluster0_1	96 (95, 98)	96 (95, 98)	96 (95, 98)	96% - 96%
10	group1_only	cluster0_2	96 (94, 97)	96 (94, 97)	96 (94, 97)	96% - 96%
10	group1_only	cluster0_only	96 (95, 98)	96 (94, 98)	96 (94, 98)	96% - 96%
10	group1_only	cluster1_2	96 (94, 97)	96 (94, 97)	95 (94, 97)	95% - 96%
10	group1_	cluster1_onl	96 (94, 98)	96 (94, 98)	96 (94, 98)	96% - 96%

	only	y				
10	group1_only	cluster2_only	94 (93, 96)	94 (93, 95)	94 (93, 95)	94% - 94%
10	group2_3	all_clusters	97 (95, 98)	97 (95, 98)	97 (95, 98)	97% - 97%
10	group2_3	cluster0_1	87 (86, 89)	86 (84, 87)	85 (84, 87)	85% - 87%
10	group2_3	cluster0_2	83 (82, 85)	82 (80, 83)	81 (80, 83)	81% - 83%
10	group2_3	cluster0_only	84 (82, 85)	82 (81, 84)	82 (80, 83)	82% - 84%
10	group2_3	cluster1_2	85 (84, 87)	83 (82, 85)	83 (82, 84)	83% - 85%
10	group2_3	cluster1_only	86 (84, 87)	84 (83, 86)	84 (82, 86)	84% - 86%
10	group2_3	cluster2_only	77 (76, 78)	82 (79, 86)	81 (79, 85)	77% - 82%
10	group2_only	all_clusters	95 (94, 97)	95 (94, 97)	95 (94, 97)	95% - 95%
10	group2_only	cluster0_1	97 (95, 98)	97 (94, 98)	97 (94, 98)	97% - 97%
10	group2_only	cluster0_2	95 (94, 97)	95 (94, 96)	95 (94, 96)	95% - 95%
10	group2_only	cluster0_only	97 (95, 98)	97 (95, 98)	97 (95, 98)	97% - 97%
10	group2_only	cluster1_2	94 (93, 96)	94 (93, 96)	94 (93, 96)	94% - 94%
10	group2_only	cluster1_only	96 (94, 98)	95 (93, 97)	95 (93, 97)	95% - 96%
10	group2_only	cluster2_only	94 (93, 95)	94 (93, 95)	94 (93, 95)	94% - 94%
10	group3_only	all_clusters	94 (92, 95)	94 (92, 95)	94 (93, 95)	94% - 94%
10	group3_only	cluster0_1	75 (72, 78)	94 (93, 96)	94 (93, 96)	75% - 94%
10	group3_only	cluster0_2	95 (94, 97)	94 (93, 96)	94 (93, 96)	94% - 95%
10	group3_only	cluster0_only	80 (75, 98)	96 (94, 98)	96 (94, 98)	80% - 96%
10	group3_only	cluster1_2	92 (91, 94)	93 (92, 95)	93 (92, 94)	92% - 93%
10	group3_only	cluster1_only	71 (64, 78)	93 (91, 94)	93 (92, 94)	71% - 93%
10	group3_only	cluster2_only	93 (92, 95)	93 (92, 95)	93 (92, 94)	93% - 93%
30	all_groups	all_clusters	83 (82, 85)	83 (81, 84)	83 (81, 84)	83% - 83%
30	all_groups	cluster0_1	73 (72, 74)	72 (71, 73)	72 (71, 73)	72% - 73%
30	all_groups	cluster0_2	70 (69, 72)	69 (68, 71)	69 (68, 70)	69% - 70%
30	all_groups	cluster0_only	70 (69, 71)	69 (68, 70)	69 (68, 70)	69% - 70%
30	all_groups	cluster1_2	71 (70, 73)	70 (69, 72)	70 (69, 72)	70% - 71%
30	all_groups	cluster1_only	71 (70, 72)	70 (69, 71)	70 (69, 71)	70% - 71%

30	all_groups	cluster2_only	65 (64, 66)	64 (63, 65)	64 (63, 65)	64% - 65%
30	group1_2	all_clusters	73 (71, 76)	94 (69, 97)	94 (69, 97)	73% - 94%
30	group1_2	cluster0_1	85 (84, 87)	84 (83, 86)	84 (83, 86)	84% - 85%
30	group1_2	cluster0_2	81 (80, 83)	80 (79, 82)	80 (79, 82)	80% - 81%
30	group1_2	cluster0_only	82 (80, 83)	81 (79, 82)	81 (79, 82)	81% - 82%
30	group1_2	cluster1_2	83 (82, 85)	82 (81, 84)	82 (81, 84)	82% - 83%
30	group1_2	cluster1_only	84 (82, 85)	83 (81, 84)	83 (81, 84)	83% - 84%
30	group1_2	cluster2_only	75 (74, 76)	74 (73, 76)	74 (73, 75)	74% - 75%
30	group1_3	all_clusters	84 (81, 86)	83 (80, 85)	83 (80, 85)	83% - 84%
30	group1_3	cluster0_1	93 (90, 96)	92 (89, 95)	92 (89, 95)	92% - 93%
30	group1_3	cluster0_2	92 (89, 94)	91 (88, 93)	90 (88, 92)	90% - 92%
30	group1_3	cluster0_only	92 (89, 95)	91 (88, 94)	91 (88, 93)	91% - 92%
30	group1_3	cluster1_2	91 (88, 93)	90 (87, 92)	90 (87, 92)	90% - 91%
30	group1_3	cluster1_only	90 (87, 93)	89 (86, 92)	89 (86, 92)	89% - 90%
30	group1_3	cluster2_only	86 (84, 88)	85 (83, 87)	85 (83, 87)	85% - 86%
30	group1_only	all_clusters	88 (85, 90)	87 (85, 90)	87 (85, 90)	87% - 88%
30	group1_only	cluster0_1	86 (84, 89)	85 (83, 88)	85 (83, 88)	85% - 86%
30	group1_only	cluster0_2	81 (79, 84)	80 (78, 83)	80 (78, 83)	80% - 81%
30	group1_only	cluster0_only	81 (79, 84)	80 (78, 83)	80 (78, 83)	80% - 81%
30	group1_only	cluster1_2	85 (82, 87)	84 (81, 87)	84 (81, 86)	84% - 85%
30	group1_only	cluster1_only	86 (84, 89)	85 (83, 88)	85 (83, 88)	85% - 86%
30	group1_only	cluster2_only	76 (74, 79)	75 (73, 78)	75 (73, 78)	75% - 76%
30	group2_3	all_clusters	86 (84, 88)	85 (83, 88)	85 (83, 88)	85% - 86%
30	group2_3	cluster0_1	76 (74, 78)	73 (71, 75)	72 (70, 75)	72% - 76%
30	group2_3	cluster0_2	92 (89, 95)	91 (88, 94)	91 (88, 94)	91% - 92%
30	group2_3	cluster0_only	66 (64, 69)	93 (62, 97)	93 (89, 96)	66% - 93%
30	group2_3	cluster1_2	71 (69, 74)	94 (66, 97)	94 (90, 97)	71% - 94%
30	group2_3	cluster1_only	74 (72, 76)	72 (70, 74)	71 (69, 74)	71% - 74%
30	group2_	cluster2_only	87 (85, 89)	86 (84, 88)	86 (84, 88)	86% - 87%

	3	y				
30	group2_ only	all_clusters	88 (85, 90)	87 (85, 90)	87 (85, 90)	87% - 88%
30	group2_ only	cluster0_1	88 (85, 90)	87 (84, 89)	87 (84, 89)	87% - 88%
30	group2_ only	cluster0_2	83 (81, 86)	83 (80, 86)	83 (80, 85)	83% - 83%
30	group2_ only	cluster0_onl y	84 (82, 87)	84 (81, 86)	84 (81, 86)	84% - 84%
30	group2_ only	cluster1_2	86 (83, 88)	85 (82, 88)	85 (82, 88)	85% - 86%
30	group2_ only	cluster1_onl y	88 (86, 90)	87 (84, 89)	87 (84, 89)	87% - 88%
30	group2_ only	cluster2_onl y	78 (76, 82)	78 (75, 81)	78 (75, 81)	78% - 78%
30	group3_ only	all_clusters	88 (85, 90)	86 (84, 89)	86 (84, 89)	86% - 88%
30	group3_ only	cluster0_1	88 (85, 90)	86 (84, 89)	86 (83, 89)	86% - 88%
30	group3_ only	cluster0_2	86 (84, 89)	85 (83, 88)	85 (82, 88)	85% - 86%
30	group3_ only	cluster0_onl y	87 (84, 89)	86 (83, 88)	85 (83, 88)	85% - 87%
30	group3_ only	cluster1_2	86 (84, 89)	85 (82, 88)	85 (82, 88)	85% - 86%
30	group3_ only	cluster1_onl y	87 (85, 89)	85 (83, 88)	85 (82, 88)	85% - 87%
30	group3_ only	cluster2_onl y	84 (82, 87)	84 (81, 87)	84 (81, 87)	84% - 84%
60	all_grou ps	all_clusters	74 (71, 77)	74 (72, 77)	74 (71, 77)	74% - 74%
60	all_grou ps	cluster0_1	74 (71, 77)	74 (72, 77)	74 (71, 77)	74% - 74%
60	all_grou ps	cluster0_2	74 (71, 77)	74 (72, 77)	74 (71, 77)	74% - 74%
60	all_grou ps	cluster0_onl y	74 (71, 77)	74 (72, 77)	74 (71, 77)	74% - 74%
60	all_grou ps	cluster1_2	74 (71, 77)	74 (72, 77)	74 (71, 77)	74% - 74%
60	all_grou ps	cluster1_onl y	74 (71, 77)	74 (72, 77)	74 (71, 77)	74% - 74%
60	all_grou ps	cluster2_onl y	74 (71, 77)	74 (72, 77)	74 (71, 77)	74% - 74%
60	group1_ 2	all_clusters	74 (71, 77)	74 (72, 77)	74 (71, 77)	74% - 74%
60	group1_ 2	cluster0_1	74 (71, 77)	74 (72, 77)	74 (71, 77)	74% - 74%
60	group1_ 2	cluster0_2	74 (71, 77)	74 (72, 77)	74 (71, 77)	74% - 74%
60	group1_ 2	cluster0_onl y	74 (71, 77)	74 (72, 77)	74 (71, 77)	74% - 74%
60	group1_ 2	cluster1_2	74 (71, 77)	74 (72, 77)	74 (71, 77)	74% - 74%
60	group1_ 2	cluster1_onl y	74 (71, 77)	74 (72, 77)	74 (71, 77)	74% - 74%
60	group1_ 2	cluster2_onl y	74 (71, 77)	74 (72, 77)	74 (71, 77)	74% - 74%

60	group1_3	all_clusters	74 (71, 77)	74 (72, 77)	74 (71, 77)	74% - 74%
60	group1_3	cluster0_1	74 (71, 77)	74 (72, 77)	74 (71, 77)	74% - 74%
60	group1_3	cluster0_2	74 (71, 77)	74 (72, 77)	74 (71, 77)	74% - 74%
60	group1_3	cluster0_only	74 (71, 77)	74 (72, 77)	74 (71, 77)	74% - 74%
60	group1_3	cluster1_2	74 (71, 77)	74 (72, 77)	74 (71, 77)	74% - 74%
60	group1_3	cluster1_only	74 (71, 77)	74 (72, 77)	74 (71, 77)	74% - 74%
60	group1_3	cluster2_only	74 (71, 77)	74 (72, 77)	74 (71, 77)	74% - 74%
60	group1_only	all_clusters	74 (71, 77)	74 (72, 77)	74 (71, 77)	74% - 74%
60	group1_only	cluster0_1	74 (71, 77)	74 (72, 77)	74 (71, 77)	74% - 74%
60	group1_only	cluster0_2	74 (71, 77)	74 (72, 77)	74 (71, 77)	74% - 74%
60	group1_only	cluster0_only	74 (71, 77)	74 (72, 77)	74 (71, 77)	74% - 74%
60	group1_only	cluster1_2	74 (71, 77)	74 (72, 77)	74 (71, 77)	74% - 74%
60	group1_only	cluster1_only	74 (71, 77)	74 (72, 77)	74 (71, 77)	74% - 74%
60	group1_only	cluster2_only	74 (71, 77)	74 (72, 77)	74 (71, 77)	74% - 74%
60	group2_3	all_clusters	74 (71, 77)	74 (72, 77)	74 (71, 77)	74% - 74%
60	group2_3	cluster0_1	74 (71, 77)	74 (72, 77)	74 (71, 77)	74% - 74%
60	group2_3	cluster0_2	74 (71, 77)	74 (72, 77)	74 (71, 77)	74% - 74%
60	group2_3	cluster0_only	74 (71, 77)	74 (72, 77)	74 (71, 77)	74% - 74%
60	group2_3	cluster1_2	74 (71, 77)	74 (72, 77)	74 (71, 77)	74% - 74%
60	group2_3	cluster1_only	74 (71, 77)	74 (72, 77)	74 (71, 77)	74% - 74%
60	group2_3	cluster2_only	74 (71, 77)	74 (72, 77)	74 (71, 77)	74% - 74%
60	group2_only	all_clusters	74 (71, 77)	74 (72, 77)	74 (71, 77)	74% - 74%
60	group2_only	cluster0_1	74 (71, 77)	74 (72, 77)	74 (71, 77)	74% - 74%
60	group2_only	cluster0_2	74 (71, 77)	74 (72, 77)	74 (71, 77)	74% - 74%
60	group2_only	cluster0_only	74 (71, 77)	74 (72, 77)	74 (71, 77)	74% - 74%
60	group2_only	cluster1_2	74 (71, 77)	74 (72, 77)	74 (71, 77)	74% - 74%
60	group2_only	cluster1_only	74 (71, 77)	74 (72, 77)	74 (71, 77)	74% - 74%
60	group2_only	cluster2_only	74 (71, 77)	74 (72, 77)	74 (71, 77)	74% - 74%
60	group3_	all_clusters	74 (71, 77)	74 (72, 77)	74 (71, 77)	74% - 74%

	only					
60	group3_only	cluster0_1	74 (71, 77)	74 (72, 77)	74 (71, 77)	74% - 74%
60	group3_only	cluster0_2	74 (71, 77)	74 (72, 77)	74 (71, 77)	74% - 74%
60	group3_only	cluster0_only	74 (71, 77)	74 (72, 77)	74 (71, 77)	74% - 74%
60	group3_only	cluster1_2	74 (73, 75)	74 (73, 75)	74 (73, 75)	74% - 74%
60	group3_only	cluster1_only	74 (73, 75)	74 (73, 75)	74 (73, 75)	74% - 74%
60	group3_only	cluster2_only	74 (73, 75)	74 (73, 75)	74 (73, 75)	74% - 74%

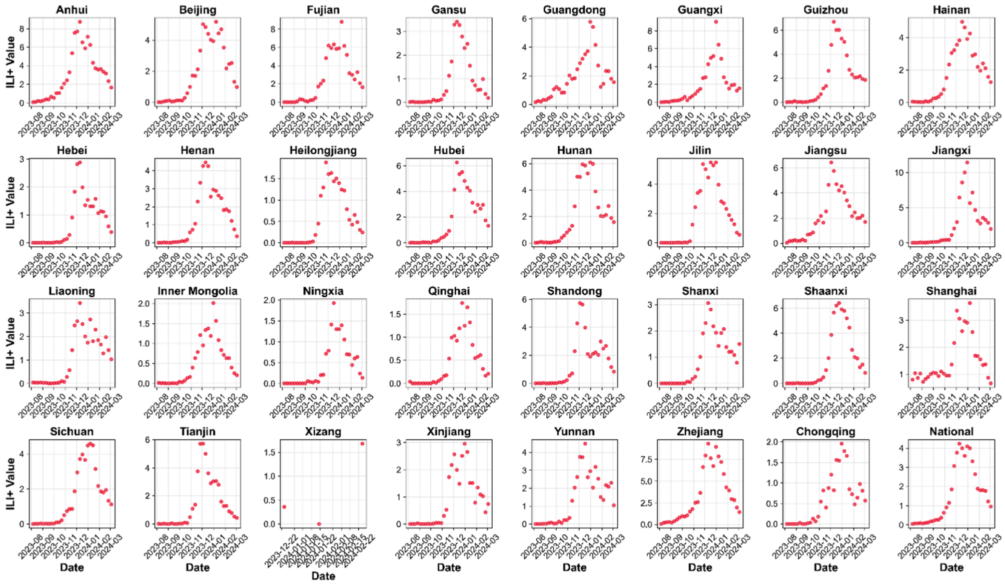

Figure S1. ILI+ scatter diagram: The horizontal axis represents dates, and the vertical axis represents the ILI+ (positive for influenza-like symptoms) value.

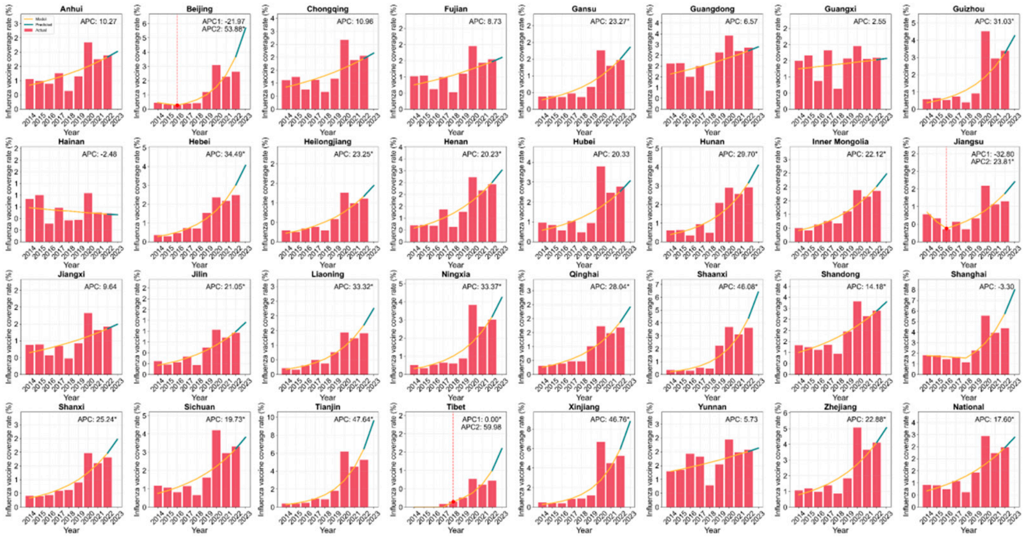

Figure S2. Annual Percent Change: The horizontal axis represents years, and the vertical axis represents influenza vaccine coverage rates. The curves in the figure illustrate annual percentage change trends. When APC> 0, it indicates an increasing trend in influenza vaccination rates, while APC <0 suggests a declining trend in influenza vaccination rates.

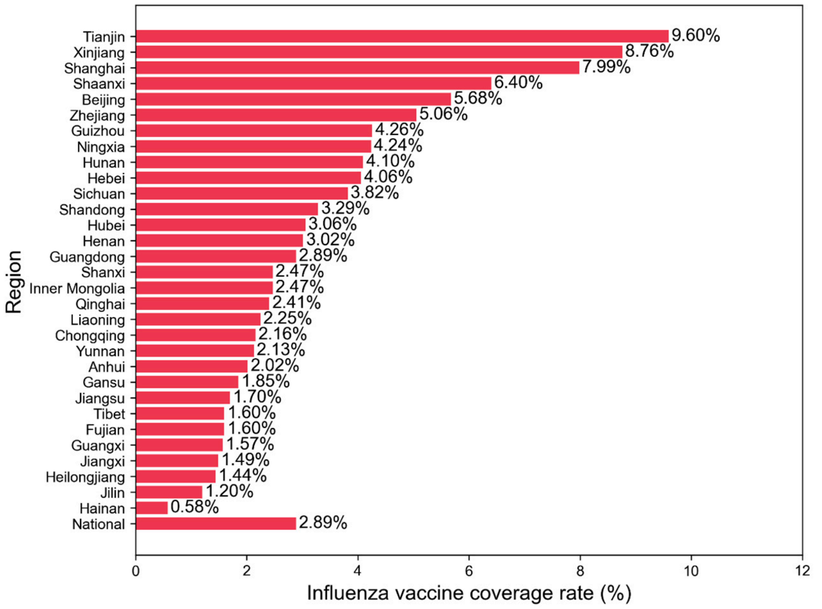

Figure S3. Vaccination coverage rates by province: The horizontal axis represents influenza vaccination coverage rate, and the vertical axis represents provinces.

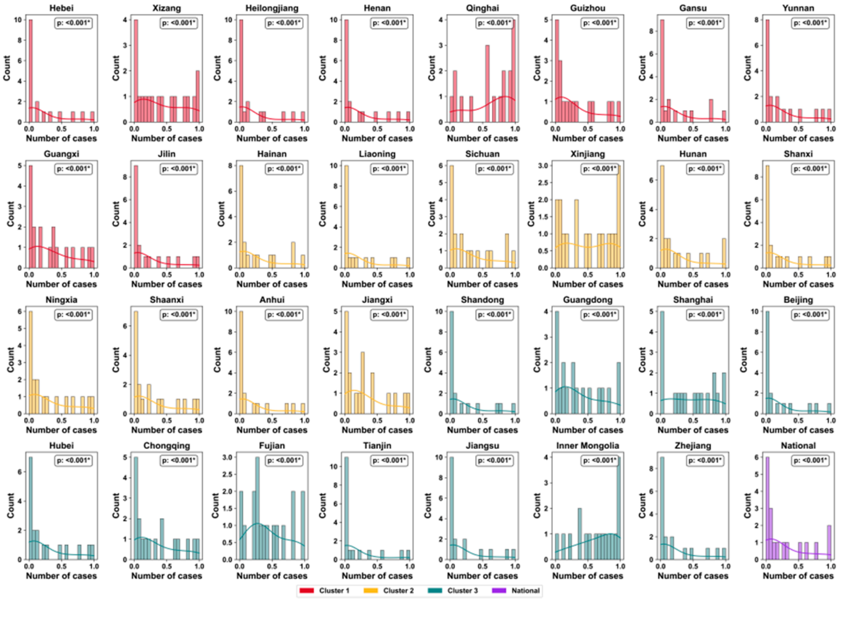

Figure S4. Simulation of Normal Distribution for Influenza Cases: The horizontal axis represents the number of influenza cases, while the vertical axis indicates sample frequency. Each plot depicts conditions across different provinces or nationwide. Distinct colors denote regional variations: red, yellow, and green correspond to Cluster 1, Cluster 2, and Cluster 3 respectively, with purple representing the national overall level. All subplots underwent normality testing, with results consistently showing p<0.001.

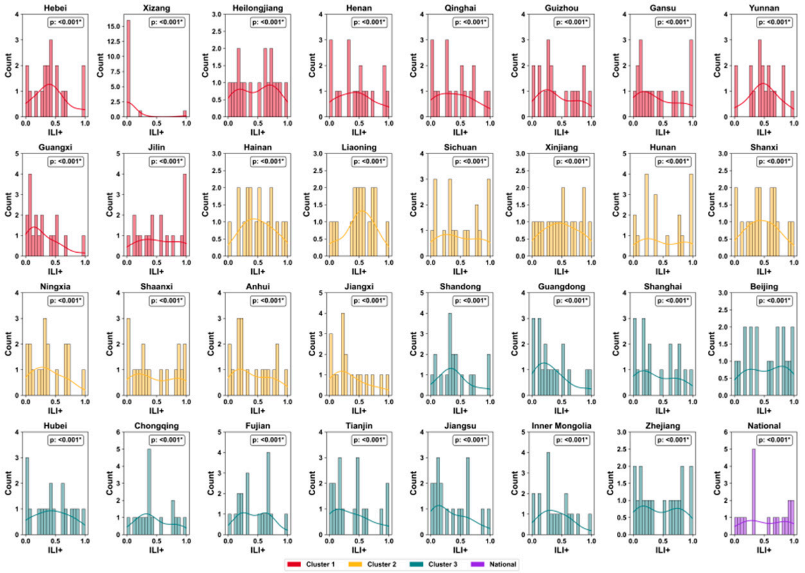

Figure S5. Simulation of Normal Distribution for Positive Index of Influenza Symptoms: The horizontal axis represents the influenza symptom positivity index, while the vertical axis indicates sample frequency. Each chart displays data distribution across different provinces or nationwide. Regional variations are represented by distinct colors: red, yellow, and green correspond to Cluster 1, Cluster 2, and Cluster 3 respectively, with purple indicating the national overall level. All subplots underwent normality testing, with consistent results showing p<0.001.

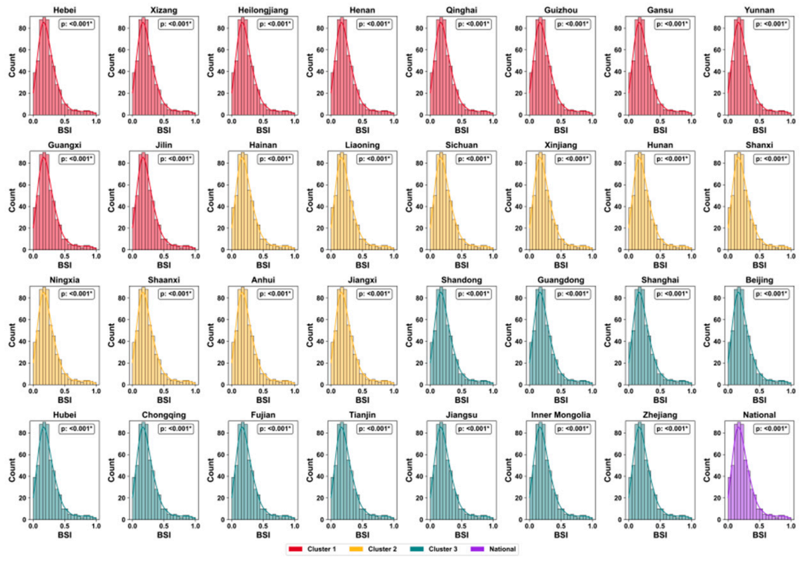

Figure S6. Normal Distribution Simulation of Baidu Influenza Search Index: The horizontal axis represents the Baidu Influenza Search Index, while the vertical axis indicates sample frequency. Each chart displays data conditions across different provinces or nationwide. Distinct colors represent regional variations: red, yellow, and green correspond to Cluster 1, Cluster 2, and Cluster 3 respectively, with purple indicating the national overall level. All subplots underwent normality testing, with consistent results showing p<0.001.

Note: The strategy names in Figures S7-S11 illustrate the combination scenarios of different strategies, with specific meanings as follows. S denotes Sequence, representing sequential vaccination strategies: S-A indicates achieving vaccination targets before the start of the epidemic season; S-B indicates that approximately 50% of the target vaccination population must be vaccinated before the epidemic season, with the remaining vaccinations conducted sequentially within the following month; S-C indicates achieving full vaccination targets during the initial outbreak phase, followed by systematic completion of the vaccination plan over the next two months. A denotes Age, representing age-specific strategies: 1, 2, and 3 represent populations under 18 years old, 18-64 years old, and 64 years and above, respectively; A-1, A-2, and A-3 indicate increased vaccination coverage within specific age groups; A-1,2, A-1,3, and A-2,3 indicate increased vaccination rates for two specific age groups; A-all denotes vaccination coverage across all age groups. R denotes Region, representing regional strategies: 0, 1, and 2 indicate underdeveloped regions, developing regions, and developed regions, respectively; R-0, R-1, and R-2 indicate increased vaccination coverage within specific regions; R-0,1, R-0,2, R-1,2 indicate increased vaccination rates for two specific age groups; R-all denotes vaccination coverage across all regions.

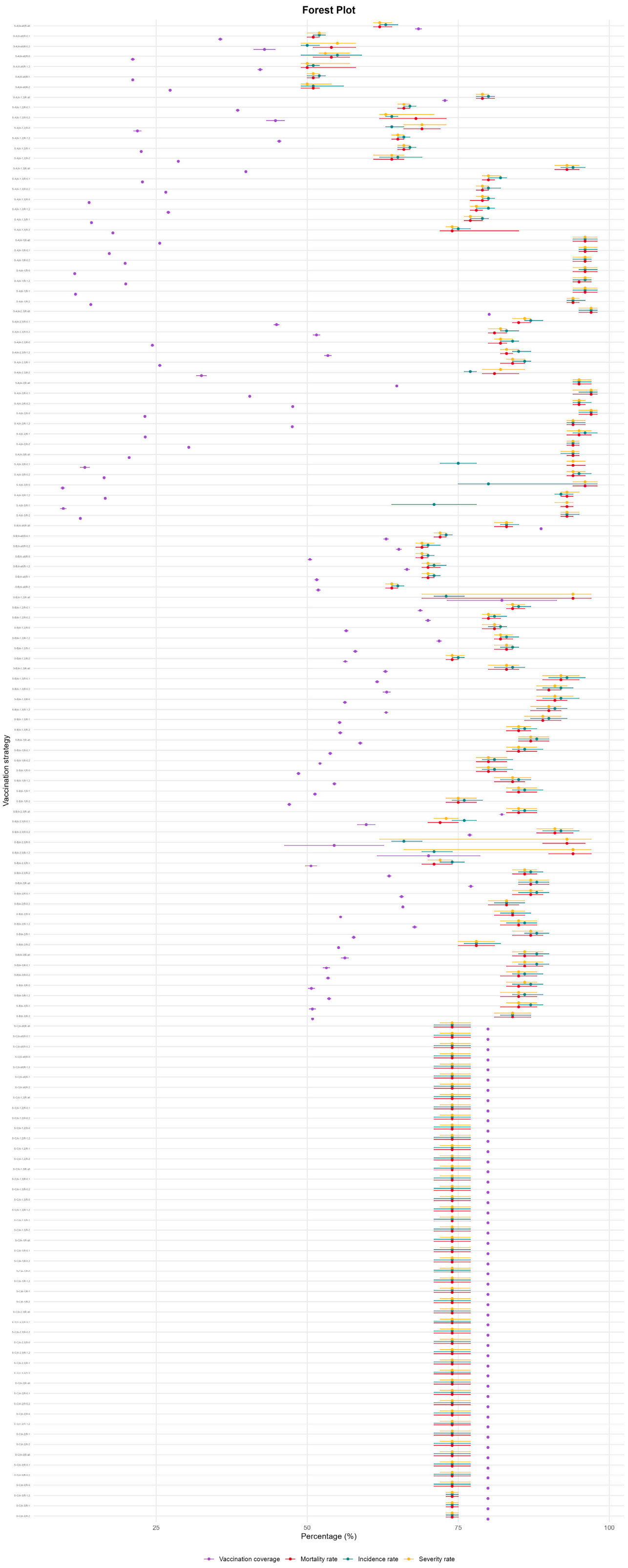

Figure S7. Forest plot of different strategy threshold values: The horizontal axis represents percentages, and the vertical axis shows different strategy names. Different colors indicate vaccination rates, incidence rates, severe case rates, and mortality rates. This figure displays the threshold values and error ranges for each strategy derived from Joinpoint regression analysis results.

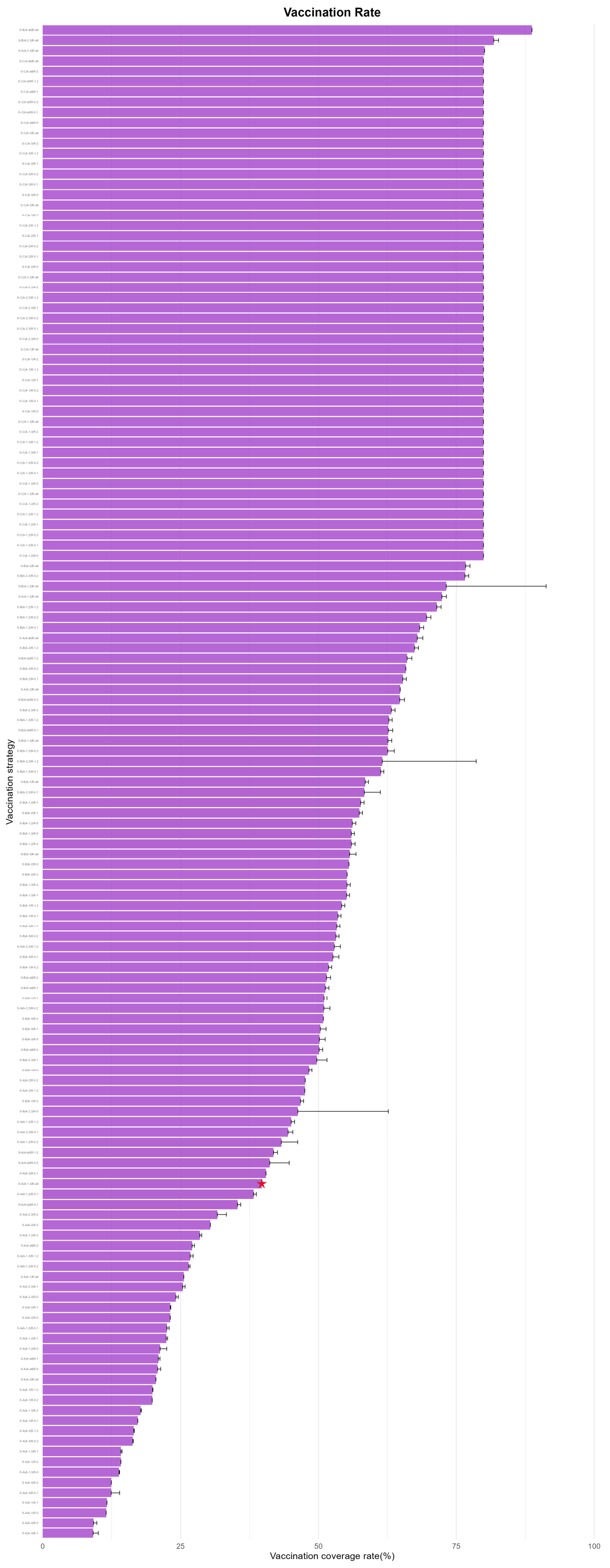

Figure S8. Threshold effect diagram of vaccination coverage rate: The horizontal axis represents vaccination rates, while the vertical axis indicates different strategies. Strategies marked with five-point stars represent the best selected strategies.

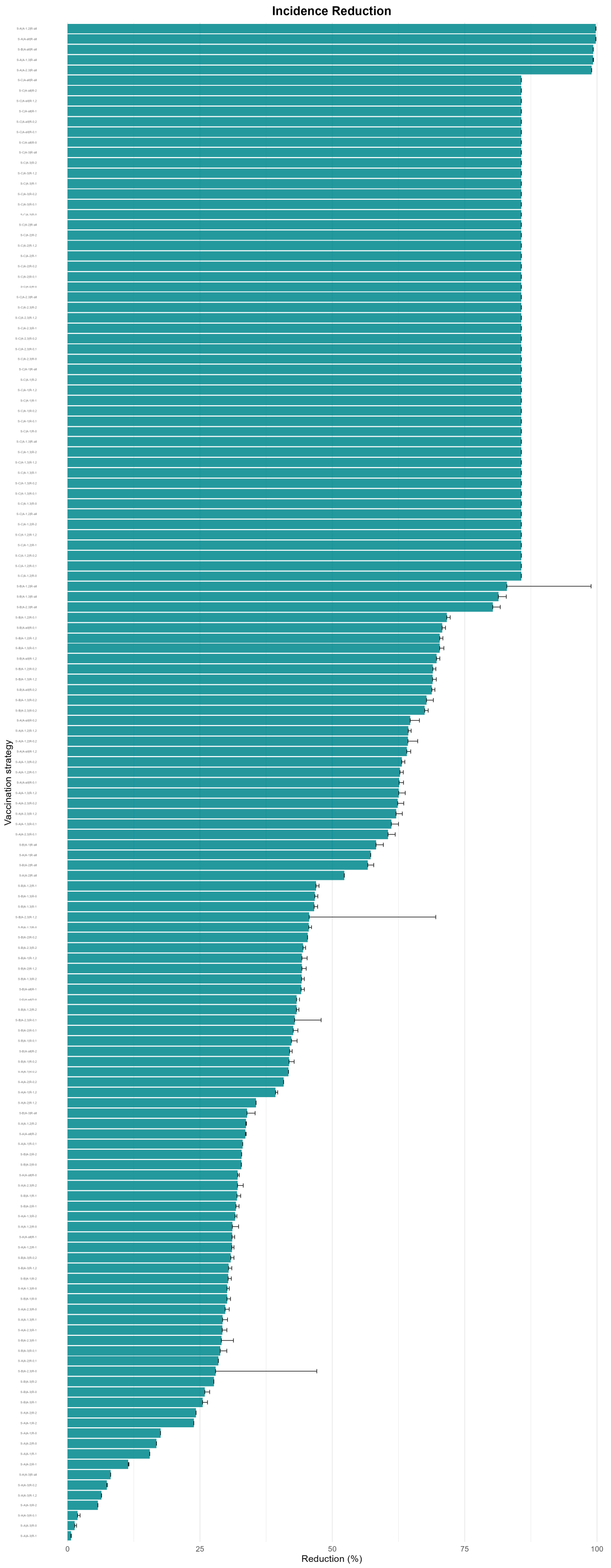

Figure S9. Threshold effect plot of incidence rate decline percentage: Threshold effect plot of percentage reduction in incidence rate: The x-axis represents the percentage reduction in incidence rate, and the y-axis represents different strategies.

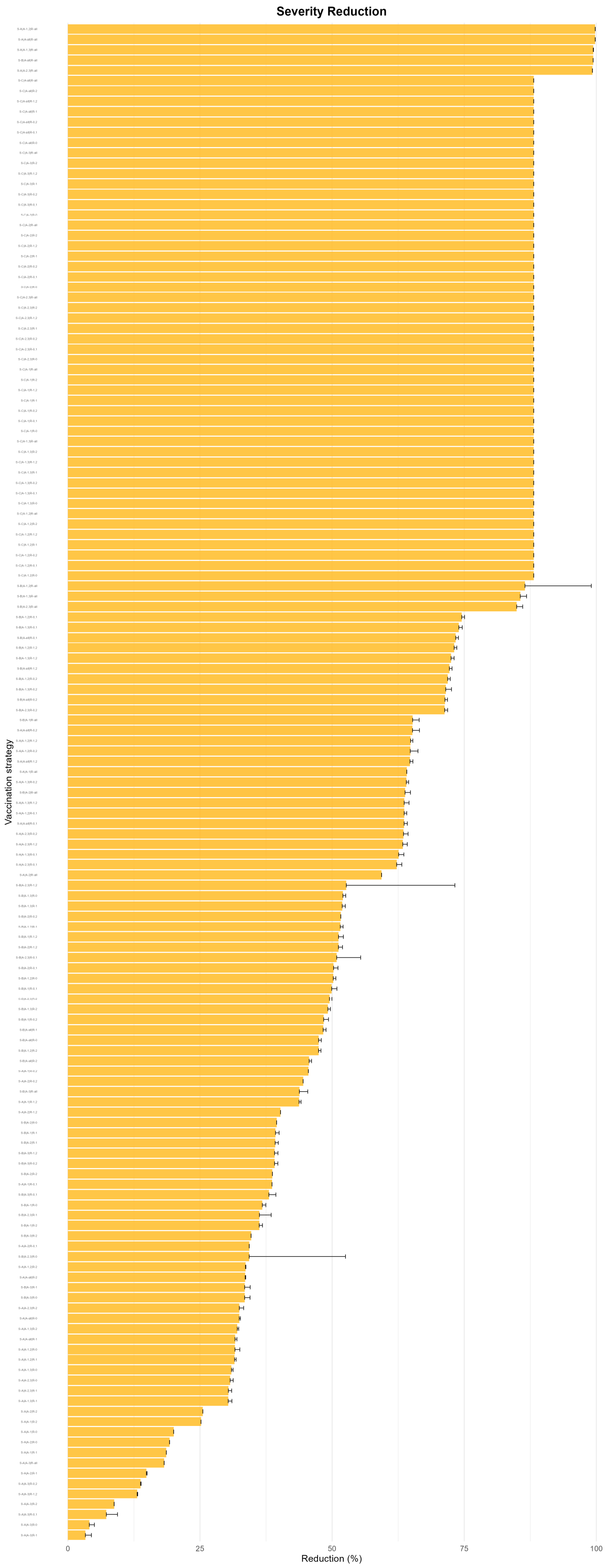

Figure S10. Threshold effect plot of severity rate decline percentage: Threshold effect plot of percentage reduction in severity rate: The x-axis represents the percentage reduction in incidence rate, and the y-axis represents different strategies.

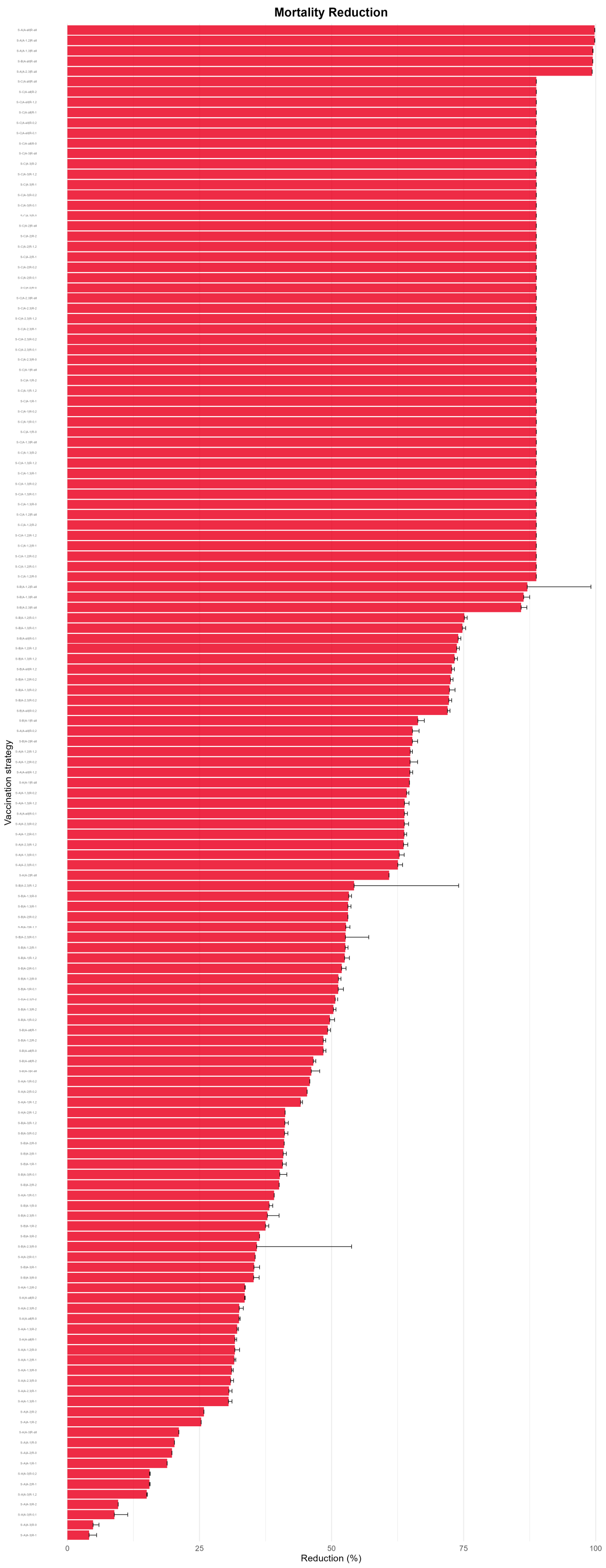

Figure S11. Threshold effect plot of mortality rate decline percentage: Threshold effect plot of percentage reduction in mortality rate: The x-axis represents the percentage reduction in incidence rate, and the y-axis represents different strategies.
